# Effect of Solvents on Lignin–Surface Interactions via Molecular Dynamics Simulations

**DOI:** 10.1021/acs.jpcb.5c02943

**Published:** 2025-08-27

**Authors:** Juriti Rajbangshi, Canan Sener, Reid C. Van Lehn

**Affiliations:** † Department of Chemical and Biological Engineering, University of Wisconsin–Madison, Madison, Wisconsin 53706, United States; ‡ DOE Great Lakes Bioenergy Research Center, University of Wisconsin−Madison, Madison, Wisconsin 53726, United States; § Department of Chemistry, University of Wisconsin−Madison, Madison, Wisconsin 53706, United States; ∥ Wisconsin Energy Institute, University of Wisconsin-Madison, Madison, Wisconsin 53726, United States

## Abstract

Lignin, an essential building block of lignocellulosic biomass, is a potential abundant source of aromatic monomers for the polymer and chemical industry. Reductive catalytic fractionation (RCF) is one promising process that can produce high yields of phenolic monomers and oligomers from lignin under different catalytic conditions. An important choice in optimizing RCF is the selection of solvent; however, detailed insights into the effects of solvent on lignin behaviors and interactions remain limited. In this work, we perform all-atom molecular dynamics simulations to study the solvation of lignin, solvent-mediated conformational changes, and the interaction of solvated lignin oligomers with model surfaces. We focus on the behavior of an oligomeric lignin model compound in methanol, ethanol, a binary mixture of ethanol and water, and water at both the RCF reaction temperature (473 K) and room temperature. Analysis of structural features of lignin suggests that these three organic solvent systems favorably solvate lignin, resulting in a more extended conformation suitable for catalytic conversion to valuable chemicals. We further introduce model palladium (Pd) and carbon (C) surfaces to understand how solvent choice impacts adsorption onto a representative catalytic surface and support and to quantify the competition among the reactant and solvent molecules for the surface. Unbiased simulations suggest strong adsorption of lignin on both Pd and C surfaces at 473 K, with notable solvent-mediated differences in adsorption energies. Additionally, our findings indicate that lignin adsorption is promoted by the entropy change resulting from the displacement of the solvent molecules from the surface. This study provides a molecular perspective of adsorption of lignin onto varying surfaces, which is a step toward understanding and optimizing the catalytic conversion of lignin into valuable chemicals.

## Graphical abstract

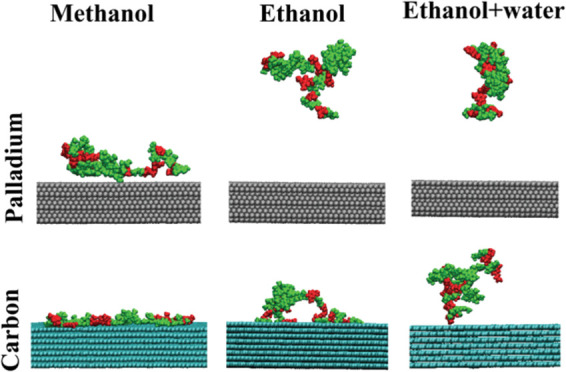

## 1 Introduction

Lignocellulosic biomass is an abundant and renewable resource which can be an alternative to petroleum feedstocks for the fuel and chemical industries.^1,2^ It primarily consists of cellulose, hemicellulose, and lignin; cellulose and hemicellulose have been extensively studied for applications in the biofuels and paper industries, while lignin can potentially be utilized to produce various valuable chemicals.^3,4^ However, challenges in lignin valorization arise from its complex heteropolymer structure^5,6^ and broad molecular weight distributions.^7–9^ Lignin is primarily composed of three types of monomers—p-coumaryl alcohol (p-hydroxyphenyl, H), coniferyl alcohol (guaiacyl, G), and sinapyl alcohol (syringyl, S)—which are primarily linked together by carbon–carbon and ether linkages.^5^ The three typical steps employed to produce valuable products from lignin are lignocellulose fractionation, lignin depolymerization, and upgrading toward the desired chemicals. These three aspects have received extensive attention in both academic and industrial research.^1,10^ For example, solvents such as γ-valerolactone,^3,11^ ammonia,^12,13^ and ionic liquids^14^ have been used for the extraction of lignin under relatively mild conditions to produce high-quality lignin streams. Numerous studies have also investigated catalytic,^5,15–17^ thermal,^18–20^ and biological approaches^21–24^ to depolymerize lignin into monomers and oligomers and upgrade the resulting products.

Most lignin depolymerization techniques use heterogeneous catalysts under either oxidative or reductive conditions^10,25,26^ to cleave β-aryl-ether bonds, which are the most common linkages and are more readily cleaved than carbon–carbon linkages.^27^ One promising approach called reductive catalytic fractionation (RCF) depolymerizes lignin from the lignocellulose matrix via hydrogenolysis^28–36^ and has been reported to produce high yields of phenolic monomers and oligomers under different catalytic conditions.^25,26^ RCF typically uses common organic solvents^28,32,35,37–39^ or aqueous solvent mixtures^31,33,34,40,41^ along with a metal catalyst.^42^ The choice of catalyst plays an important role in determining RCF yields and products, as the hydrogenolysis of C–O bonds is metal-dependent.^2,10,43,44^ The most common heterogeneous catalysts used are Rh, Pd, Ru, and Ni supported on activated C or Al_2_O_3_, which favor products containing syringyl (S), guaiacyl (G), and p-hydroxyphenyl (H) phenolic substructures with propanol side chains.^26,45–49^ These product mixtures show promise for further upgrading; for example, RCF in an alcohol solvent over a heterogeneous Pd/C catalyst has been found to favor the generation of product mixtures that can be microbially converted to valuable products at high yield.^50^

In addition to the selection of catalyst, the selection of solvent can also alter the distribution of products and reaction yields from RCF, motivating the study of a wide range of solvents for RCF.^10,51^ Common organic solvents such as methanol, ethanol, 2-propanol, ethylene glycol, dioxane and aqueous mixtures of organic solvents have been used as solvents in the reductive depolymerization of lignocellulose.^37,38,52^ Methanol and ethylene glycol were reported to produce high yields of lignin monomers and dimers during an RCF process.^37^ Aqueous solvent mixtures such as methanol + water and ethanol + water were also reported to enhance lignin conversion.^40^ However, there is a lack of molecular-scale insight regarding how solvent selection impacts lignin behaviors and interactions. Past studies of heterogeneous catalysis have shown that the solvent environment can significantly influence key aspects of catalysis, such as site-specific catalytic activity^53^ and selectivity.^28,54^ Solvent molecules can also compete with reactants and/or products for the active sites on the catalyst surface.^55^ This competition highlights the need to understand how solvent selection affects the accessibility of lignin to the catalyst surface. Earlier studies have demonstrated that solvents such as methanol and its aqueous mixtures can lead to extended coil conformations of lignin, which are referred to as “catalytically competent” conformations.^56^ These conformations expose linkages to the surrounding solvents, thereby facilitating depolymerization. Recent work on plastic recycling has similarly demonstrated that polymer chain organization, and resulting interactions with the catalytic surface, is crucial for determining adsorption and reactivity.^57^ These considerations motivate our study of the solvation of lignin, the interaction of solvated lignin oligomers with catalytically relevant surfaces, and the structural changes of lignin in different solvent environments.

In this work, we perform all-atom molecular dynamics (MD) simulations of oligomeric lignin model compounds in various organic solvents, including methanol, ethanol, a binary mixture of ethanol + water (85:15, v:v), and water at both a typical RCF reaction temperature (473 K) and room temperature. Such simulations have previously been used to reveal solvent effects relevant to biomass conversion and lignin behavior.^56,58–69^ We investigate conformational changes of the lignin in these solvent systems by analyzing structural features, such as the radius of gyration and solvent-accessible surface area of lignin. These analyses indicate that the three organic solvents can better solvate lignin than water, resulting in a more extended configuration suitable for the catalytic conversion to valuable chemicals. We further introduce the presence of either a model Pd or carbon (C) surface to understand how the choice of solvent impacts adsorption onto a typical catalyst surface or support and to quantify the competition among the reactants and solvents for the surface. Unbiased simulations suggest that there is strong adsorption of lignin on both Pd and C surfaces at 473 K. To further quantify adsorption thermodynamics, we computed adsorption energies by employing a previously reported and computationally efficient approach,^5^ which involves separately calculating contributions due to lignin-solvent, lignin-solvent-surface, solvent-surface, and solvent–solvent interactions. Analysis of adsorption energies indicates notable solvent-mediated differences in adsorption energy, with further analysis of the solvent structure indicating that lignin adsorption is also driven by the entropy gain associated with the liberation of solvent molecules from the surface. These conclusions are further validated by adsorption free energy calculations for the individual monomers. Overall, our study provides a molecular perspective of adsorption of lignin onto Pd and C surfaces, which is a step toward understanding and optimizing the catalytic conversion of lignin into valuable chemicals.

## 2 Methods

### 2.1 Solvent Systems and Lignin Oligomer Selection

We performed unbiased all-atom MD simulations of oligomeric lignin model compounds in three different organic solvents and water. We chose solvent systems and conditions for our simulations corresponding to available experimental reports on RCF,^50^ where methanol in combination with a Pd/C catalyst and H_2_ was utilized at 30 bar and 473 K. We thus studied methanol and ethanol as two possible alcohols. Because binary mixtures of organic solvents with water have been reported to improve lignin solubilities,^40,70^ we also considered a mixture of ethanol and water (85:15 v:v), which we refer to as ethanol + water. Finally, pure water was chosen as a representative poor solvent for lignin. Simulations were performed at both the RCF reaction temperature (473 K) and room temperature (298 K). Organic solvents were parametrized using the CGenFF/CHARMM36 force fields,^71,72^ whereas the TIP4P/2005 model^73^ was used for water.

Structural models of lignin oligomers were generated by considering available experimental reports on poplar lignin biomass (a*Populus maximowiczii* × *nigra* hybrid, NM6), which indicate that the ratio of syringyl/guaiacyl lignin subunits (i.e., S/G ratio) of poplar lignin extracted using the GVL process is 2.12 (S:G 68:32)^50^ and peak of the molecular weight distribution is ∼5700 Da.^74^ We selected a set of five lignin oligomers with S/G ratios between 1.55 and 2.25 and molecular weights in the range of 2500–5900 Da to mimic these values. Representative structures were obtained using the LigninBuilder software^75^ by filtering the Birch library to select four structures (denoted as B1–B4 in Table 1) and filtering the Miscanthus library to obtain one structure (denoted as M5 in Table 1) inside this software. Schematic representations and structural details of these lignin structures are provided in Figure 1 and Table 1, respectively. The oligomeric structures obtained from LigninBuilder were then used to generate the force field parameters from the CHARMM-based lignin force field.^76^ Force field parameters and GROMACS-compatible input files were prepared using the TopoTools plugin implemented in VMD,^77^ in combination with the TopoGromacs script.^78^

**Figure 1:**
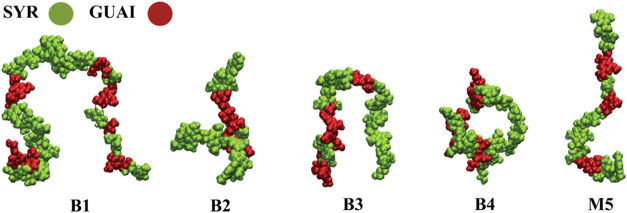
All-atom structures of lignin oligomers colored by their syringyl or guaiacyl subunits. Oligomer labels indicate whether structures were obtained from the Birch (B) or Miscanthus (M) LigninBuilder libraries, as described in Table 1.

**Table 1:** Number of S/H/G (Syringyl/p-Hydroxyphenyl/Guaiacyl) Lignin Subunits, Interunit Linkages, and Molecular Weights (MWs) for the Selected Lignin Oligomers

	S	H	G	β–*O*–4	β–5	5–5	4–*O*–5	β–1	α–*O*–4	β–β	MW (Da)
B1	19	0	9	20	0	1	2	1	1	2	5867.99
B2	9	0	4	7	1	0	0	1	2	1	2644.74
B3	10	0	5	11	1	0	1	0	1	0	3157.24
B4	14	0	9	14	0	1	2	3	2	0	4626.74
M5	8	0	4	7	3	0	0	0	0	1	2506.57

Each simulation system was prepared by placing a single lignin oligomer (one of B1–B4 or M5 in Table 1) in a simulation box and solvating it with respective solvents using the *gmx solvate* tool in GROMACS. The number of solvent molecules was determined based on the box dimensions and the van der Waals volume of the solute (in this case, a lignin oligomer). Box sizes were chosen to ensure adequate solvation and to avoid artifacts due to periodic boundary conditions. For the ethanol + water mixture, the relative number of ethanol and water molecules was calculated based on the experimental volume fraction (85:15, v:v).

### 2.2 Modeling Palladium and Carbon Surfaces

We utilized the CHARMM-GUI Nanomaterial Modeler^79,80^ to construct model Pd(111) and carbon surfaces. We employed the Interface Force Field (IFF)^81^ to describe both the surfaces and their interactions with lignin oligomers in organic solvents. This force field has been previously validated for metal–organic and metal–electrolyte interfaces and is fully compatible with CHARMM/CGenFF. Of relevance to this study due to the aromatic rings present in the lignin subunits, IFF parameters have been validated for the adsorption of benzene/naphthalene on Pd(111) and Pt(111) surfaces,^82^ thus providing a chemically relevant framework for simulating lignin-Pd/C interactions in the presence of different solvents. For the oligomer adsorption calculations, Pd was modeled as a 3D periodic slab with a Pd(111) surface of area 9.08 × 8.10 nm^2^ facing the solvent and a thickness of 2.02 nm. The area of the Pd surface spanned the entire *xy*-plane simulation box, which had a total height of 13.02 nm along the *z-*axis when including solvent. The C surface was modeled as graphite using the Carbonaceous Nanomaterials module of the Nanomaterial Modeler with a box-spanning area of 9.37 × 8.12 nm^2^, a thickness of 2.68 nm, and a total simulation system height of 13.68 nm when including the solvent. We selected 11.0 nm as the distance from the surface to the top of simulation box to ensure the lignin oligomer only interacted with one side of each 3D slab. The lignin oligomer was randomly inserted into the simulation box using the *gmx insert-molecules* tool and solvated using the *gmx solvate* tool. Figure S1 shows a schematic representation of two systems, one containing lignin on a Pd surface in methanol and the other containing lignin on a C surface in methanol.

### 2.3 Molecular Dynamics Simulation Parameters

All-atom molecular dynamics (MD) simulations were performed using Gromacs 2021.5.^83^ Simulations were performed with a single lignin oligomer solvated with either pure solvents or a binary mixture of solvents. Each system was first energy minimized using the steepest descent algorithm with a maximum of 5000 steps, a maximum step size of 0.01 nm, and a maximum tolerance of 10 kJ mol^–1^ nm^–1^. Following minimization, systems were equilibrated in the canonical (NVT) ensemble for 100 ps and then further equilibrated for 10 ns in the isothermal–isobaric (NPT*)* ensemble. The temperature and pressure were coupled using a velocity-rescaling thermostat^84^ and isotropic Berendsen barostat^85^ with 2.0 and 4.0 ps time constants, respectively. The isothermal compressibility for the barostat was 5 × 10^–5^ bar^–1^. Production simulations were performed for 500 ns by using the *NPT* ensemble. All simulations used a 2 fs time step. Configurations from the simulation trajectories were saved every 10 ps. These simulations were performed at 1 bar and either 298 or 473 K. Bonds involving hydrogen atoms were constrained using the LINCS algorithm.^86^ Verlet neighbor lists were generated using a 1.2 nm neighbor list cutoff. van der Waals interactions were modeled with a Lennard-Jones potential using a spherical cutoff distance of 1.2 nm. Long-ranged electrostatic interactions were calculated using Particle Mesh Ewald (PME)^87^ algorithm with a short-range cutoff of 1.2 nm, grid spacing of 0.12 nm, and fourth-order interpolation. We used VMD to visualize simulation configurations.^88^

### 2.4 Oligomer Adsorption Energy Simulations

We computed adsorption energies for lignin on Pd and C surfaces by utilizing an approach developed by Heinz et al.,^89^ which has previously been applied for the efficient calculation of peptide adsorption energies on the (111) and (100) surfaces of Pd, Au, and mica.^89,90^ This approach involves simulating four independent systems, containing: (1) lignin, surface, and solvent; (2) lignin and solvent; (3) solvent only; and (4) surface and solvent. All four simulations are performed in systems containing equal molecular volumes for each component. Additional system information, including box dimensions and the number of solvent molecules, is provided in Tables S1 and S2. Equilibration for these simulations followed the same protocols described in Section 2.3, with the exception of the application of anisotropic pressure coupling for the systems with Pd and C surfaces to permit only the *z*-dimension of the simulation box to vary during *NPT* simulations. Production simulations for the systems containing lignin, the surface, and solvent were performed for 500 ns, whereas production simulations for all other systems were performed for 100 ns. The time evolution of the energies for representative systems is presented in Figure S2 to demonstrate that the chosen simulation time is sufficient.

The adsorption energy is calculated via eq 1:

1 $$E_{\mathrm{ads}}=E_{1}-E_{2}+E_{3}-E_{4}$$

where *E*_1_, *E*_2_, *E*_3_, and *E*_4_ correspond to the ensemble-average energies for each of the four systems listed above. This approach accounts for the interactions between lignin and the surface, lignin and the solvent, solvent and the surface, and the solvent with itself, as illustrated in Figure S3. Eq 1 systematically subtracts interaction terms to ensure that the adsorption energy (*E*_ads_) reflects only the energy contribution from the interaction between the lignin oligomer and the surface. Figure 2 provides an illustrative example of the calculation of *E*_ads_ for lignin on Pd in ethanol + water at 473 K. *E*_ads_ is calculated by computing the energy in the lignin-solvent-surface system (*E*_1_), subtracting interactions in the lignin-solvent system (*E*_2_), adding back interactions in the pure solvent system (*E*_3_), and finally subtracting interactions in the solvent-surface system (*E*_4_); note that adding *E*_3_ accounts for subtracting solvent–solvent interactions in both *E*_1_ and *E*_4_. By maintaining the same volume of solvent throughout this process, *E*_ads_ represents the interaction between lignin and the surface in the presence of the solvent. The standard errors of the energies *E*_*i*(*i*=1,2,3,4)_ in the individual simulations were obtained by block averaging over a number of segments of the total simulation trajectory (detailed in section S3.1). The standard error for *E*_ads_ was determined by error propagation based on the standard errors of the individual system energies (detailed in the section S3.2). Adsorption energies are reported from two independent replicas of the entire simulation workflow (*i*.*e*., replicas of all four systems) using different initial configurations.

**Figure 2:**
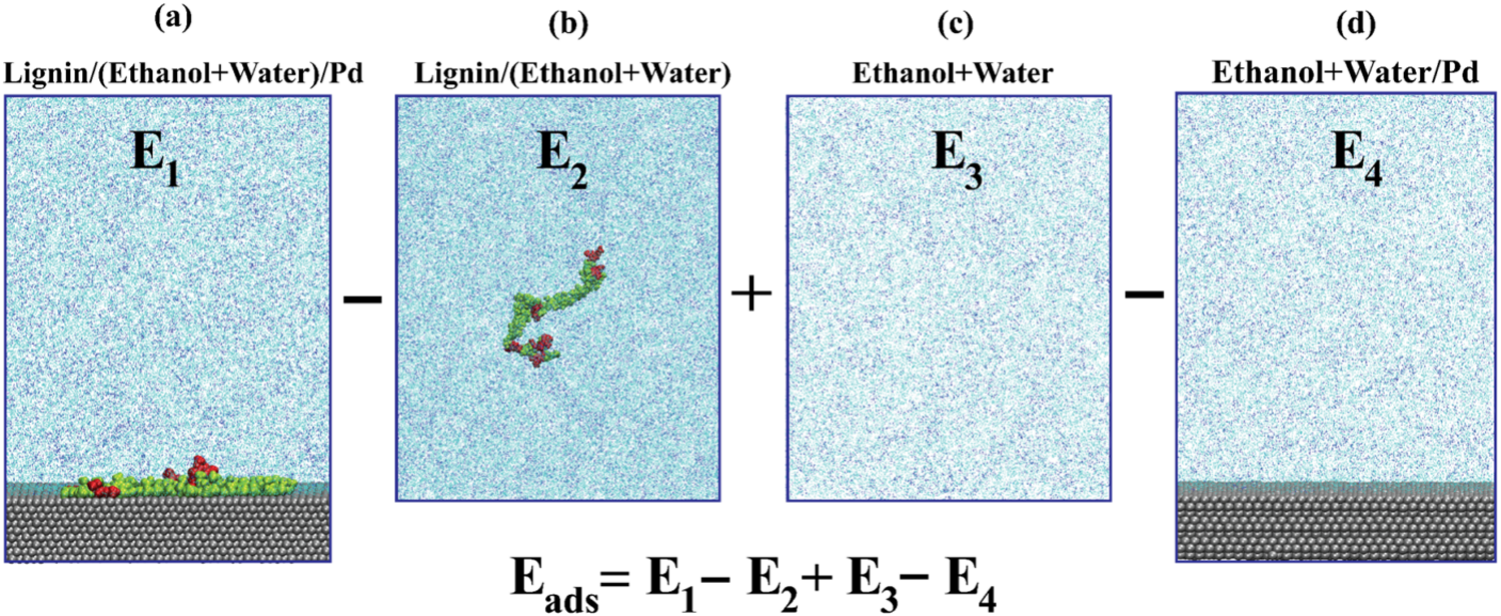
Schematic representation of the adsorption energy (*E*_ads_) calculation for lignin on the Pd surface in ethanol + water at 473 K. The adsorption energy is calculated using the equation *E*_ads_ = *E*_1_ – *E*_2_ + *E*_3_ – *E*_4_, where *E*_1_ is the average energy of the lignin-solvent-Pd surface system (a), *E*_2_ is the average energy of the lignin-solvent system (b), *E*_3_ is the average energy of the pure solvent system (c), and *E*_4_ is the average energy of the solvent-Pd surface (d).

### 2.5 Monomer Adsorption Free Energy Simulations

Umbrella sampling simulations were performed at 473 K (the RCF reaction temperature) to compute potentials of mean force (PMFs) and thereby evaluate the free energy of adsorption of a single lignin monomer (either syringyl or guaiacyl) onto the Pd surface in methanol, ethanol, and ethanol + water. Each system consisted of one lignin monomer, a 3D periodic slab of a Pd(111) surface, and the solvents. The system size was reduced compared to the oligomer adsorption simulations for computational efficiency; details are in SI Section S1. Force field parameters were consistent with those described in Section 2.3. Initial configurations for umbrella sampling were generated using steered MD with a pulling rate of 0.003 nm ps^–1^ and a spring constant of 3000 kJ mol^–1^nm^–2^. The reaction coordinate for both steered MD and umbrella sampling simulations was defined as the *z*-component of the distance (denoted as *d*_*z*_) between the center-of-mass (COM) of the monomer and the COM of the top layer of the Pd surface. A total of 31 umbrella windows were spaced at 0.05 nm intervals along *d*_*z*_, covering the range from 0.5 nm (the adsorbed state) to 2.0 nm (the desorbed state). A spring constant of 15,000 kJ mol^–1^ nm^–2^ was applied for windows in the range *d*_*z*_ = 0.50–0.75 nm to ensure adequate sampling near the tightly bound region. For the remaining windows, a spring constant of 5000 kJ mol^–1^ nm^–2^ was used. Each window was sampled for 30 ns in the NPT ensemble. The first 15 ns of each trajectory were discarded as equilibration, and the last 15 ns were used to compute PMF using the weighted histogram analysis method (WHAM).^91^ The final PMFs were shifted such that the average PMF value in the region 1.8 nm < *d*_*z*_ < 2.0 nm was equal to zero, corresponding to the desorbed reference state.

## 3 Results and Discussion

### 3.1 Effect of Solvents on Lignin Structure in Bulk Solution

The overall goal of this study is to elucidate the effect of varying solvents on the lignin behavior in bulk solution and at the surface of a model catalyst or its support. It is well known that polymer solvation can be understood by assessing solvent quality, which connects the relative strength of intrapolymer and polymer–solvent interactions to the conformation that the polymer adopts in solution.^92^ In a good solvent, the polymer adopts an extended conformation due to favorable interactions between polymer and solvent, whereas in a poor solvent, the polymer adopts a collapsed structure due to intrapolymer interactions and less favorable polymer–solvent interactions. As an initial approach to predict the quality of the solvents studied in this work, we characterized each solvent system using Hansen Solubility parameters (HSPs) as chemical descriptors of solvent properties.^93^ The three HSPs quantify the strength of dispersion interactions (δ_*D*_), dipole–dipole interactions (δ_*P*_), and hydrogen bonding interactions (δ_H_) for both a solvent and solute (*i*.*e*., lignin). By estimating HSPs for lignin, we can then compute relative energy difference (RED) values to assess the affinity between lignin and each solvent system, as detailed in the SI, with lower RED values indicating more favorable solvation and a threshold RED value of 1 distinguishing between good and poor solvents. Several prior studies have similarly applied HSP theory to screen and predict solvents for effective lignin solvation.^94–96^ Our analysis (Table S3) shows that ethanol and ethanol + water have similar RED values of ∼0.85, and hence should both be good solvents for lignin while exhibiting similar solvation behavior. Methanol has a RED value of 1.03, which is sufficiently close to the threshold that it is also expected to be a good solvent, although will likely not solvate lignin as favorably as ethanol or ethanol + water. Water has a RED value of 1.83 and, hence, is expected to be a poor solvent. These calculations provide baseline expectations for solvent quality to motivate further MD investigation of lignin conformations in solution and at surfaces.

We first sought to quantify the effect of different solvent environments on the lignin structure by analyzing the radius of gyration (*R*_g_) and solvent-accessible surface area (SASA) for each lignin oligomer. These structural descriptors were computed using the *gmx gyrate* and *gmx sasa* tools available in GROMACS. The SASA was specifically calculated using the grid-based Double Cubic Lattice Method (DCLM).^97^ The *R*_g_ and SASA can distinguish conformational differences associated with good and poor solvents to validate expectations based on HSPs; extended conformations in good solvents are associated with larger *R*_g_ and SASA values than collapsed conformations in poor solvents. Average *R*_g_ and SASA values for all lignin oligomers were computed by sampling simulation configurations starting from the time point in the production trajectories at which these values reached a consistent plateau (*i*.*e*., after equilibration), which we defined as 200 ns. Figures S4 and S5 show representative plots of *R*_g_, SASA, and system volume versus simulation time to demonstrate equilibration by this time. Table 2 shows average *R*_g_ and SASA values for all five lignin oligomers at 298 K. To demonstrate the effect of temperature on lignin structure, Figure 3 shows the average *R*_g_ and SASA values for one representative oligomer (B1) at both 298 and 473 K. At 298 K, both values are similar in methanol, ethanol, and ethanol + water, whereas lower values are observed in pure water. As expected based on the HSP analysis, these results indicate that the organic solvents all act as good solvents, leading to more extended lignin conformations, whereas pure water is a poor solvent, leading to a collapsed structure. Figure S7 shows simulation snapshots of these structures for the B1 lignin oligomer. The similar behavior of ethanol and ethanol + water, despite the addition of the poor solvent (water) to the mixture, agrees with HSP predictions and indicates the suitability of HSPs for predicting bulk solvation behavior aligned with MD results. To confirm the observed similarities for ethanol and ethanol + water in the MD simulations, Figure S9 shows radial distribution functions, which indicate that the ethanol + water mixture yields a similar solvation environment to pure ethanol.

**Figure 3:**
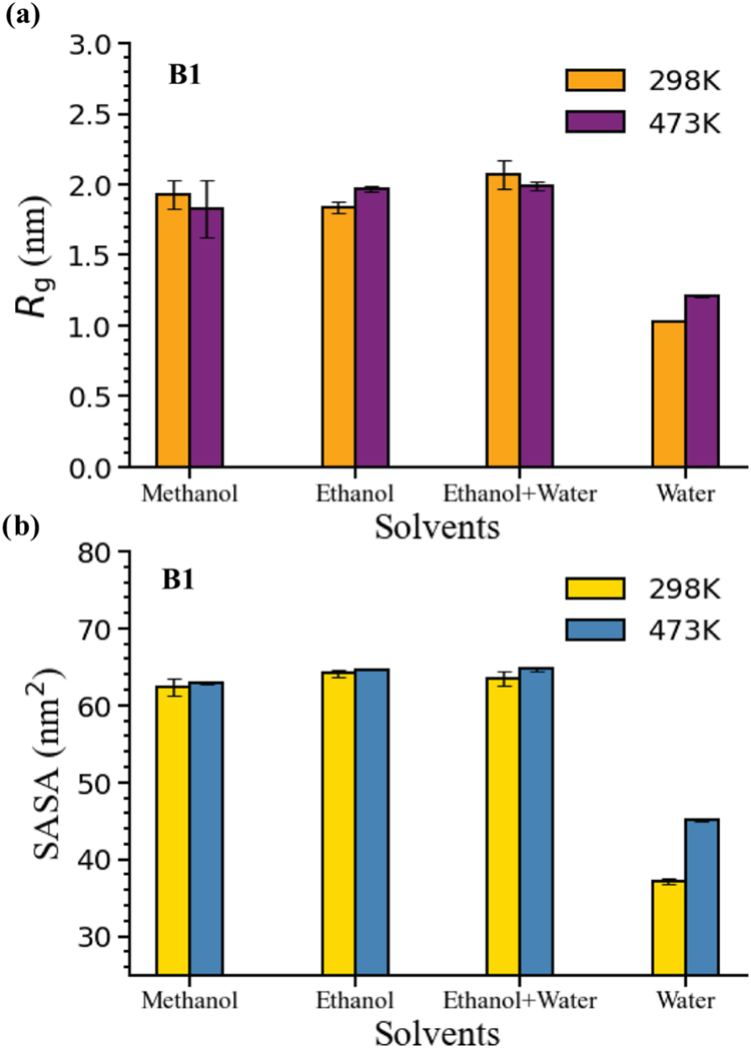
Analysis of the lignin structures in bulk solution. Two simulation quantities are computed for the B1 lignin oligomer at 298 and 473 K: (a) the average radius of gyration (*R*_g_) and (b) the solvent-accessible surface area (SASA). Average *R*_g_ and SASA values for other oligomers at 298 K are also provided in Table 2. Error bars represent the block-averaged standard error (method detailed in Section S3.1). Averages and errors were calculated from the 200–500 ns interval of the production trajectory. The corresponding standard deviation estimated from the same trajectory (see Figure S6), reflecting structural fluctuations of lignin in different solvents, is reported in Figure S4.

**Table 2:** Average Radii of Gyration (*R*_g_) and SASA Values for All Lignin Oligomers in All Solvents at *T* = 298 K^a^,^b^

property	oligomers	methanol	ethanol	ethanol + water	water
*R*_g_ (nm)	B1	1.93 ± 0.1 (0.34)	1.84 ± 0.04 (0.23)	2.07 ± 0.1 (0.37)	1.03 ± 0.004 (0.01)
	B2	1.05 ± 0.01 (0.09)	1.06 ± 0.15 (0.09)	1.01 ± 0.02 (0.09)	0.85 ± 0.01 (0.05)
	B3	1.30 ± 0.05 (0.22)	1.38 ± 0.04 (0.22)	1.39 ± 0.04 (0.21)	0.85 ± 0.02 (0.02)
	B4	1.68 ± 0.03 (0.23)	1.53 ± 0.04 (0.24)	1.51 ± 0.04 (0.23)	0.99 ± 0.01 (0.05)
	M5	1.33 ± 0.04 (0.23)	1.42 ± 0.07 (0.26)	1.29 ± 0.05 (0.21)	0.80 ± 0.01 (0.03)
SASA (nm^2^)	B1	62.38 ± 1.12 (4.6)	64.14 ± 0.42 (3.6)	63.54 ± 0.90 (4.0)	37.15 ± 0.35 (0.96)
	B2	29.26 ± 0.15 (2.0)	29.43 ± 0.35 (2.0)	29.62 ± 0.42 (2.0)	22.67 ± 0.14 (0.99)
	B3	34.87 ± 0.31 (2.7)	35.59 ± 0.4 (2.9)	35.39 ± 0.28 (2.3)	25.41 ± 0.18 (0.87)
	B4	49.84 ± 0.33 (2.9)	48.81 ± 0.67 (3.0)	48.53 ± 0.9 (4.0)	32.01 ± 0.4 (1.78)
	M5	29.87 ± 0.35 (2.1)	30.37 ± 0.47 (2.3)	29.61 ± 0.39 (1.8)	21.02 ± 0.12 (0.86)

a Uncertainties in average *R*_g_ and SASA values represent the block-averaged standard error (detailed in Section S3.1) calculated over the 200–500 ns portion of the production trajectory.

b Uncertainties shown in parentheses indicate the standard deviation over the same trajectory.

Figure 3 also shows that the average *R*_g_ and SASA values for the B1 oligomer are similar at both temperatures for all solvents except water, in which both quantities had higher values at 473 K. These results indicate that there is no substantial temperature-dependent change in the lignin structure in the bulk solution. The *R*_g_ histograms for the B1 oligomer at both temperatures, as shown in Figure S11, further confirm this trend. To further characterize factors that influence temperature-dependent variations in lignin conformations, we analyzed the time-averaged number of hydrogen bonds between lignin and the solvent (Figure S10). This analysis shows that with increasing temperature, the number of hydrogen bonds decreases across all solvents, as expected due to thermal disruption of hydrogen bonding at the high RCF temperature. However, the *R*_g_ and *SASA* values remained nearly unchanged across temperatures (Figure 3), suggesting that the apparent insensitivity of lignin conformation arises from a balance between thermal perturbation and weakened hydrogen bonding. Table 2 indicates that all lignin oligomers (B2, B3, B4 and M5) exhibit similar solvation behavior trends as B1 in all solvents; consequently, we will focus only on the B1 oligomer for the rest our analyses because it has an S/G ratio of 2.11 and a molecular weight of 5868 Da (Table 1) that closely match the experimentally reported S/G ratio of 2.12 and the peak of the molecular weight distribution at ∼5700 Da for GVL-extracted poplar lignin.^50,74^ Overall, these results indicate that the three organic solvent systems can effectively solvate lignin at both temperatures. The extended conformation observed in these good solvents suggests that they should promote the catalytic conversion of lignin by exposing reactive sites to the solvent, although the *R*_g_ and SASA analyses show no significant qualitative difference among the three organic solvents in bulk solution.

### 3.2 Interactions of Lignin with Model Surfaces

#### 3.2.1 Effect of Surfaces on Lignin Structures

As the *R*_g_ analysis demonstrated the ability to qualitatively differentiate between good and poor solvents for lignin in solution, we extended our investigation by introducing model Pd and C surfaces to determine the impact of surface interactions on lignin conformations. Simulations were performed with the same B1 lignin oligomer solvated in methanol, ethanol, and ethanol + water (*i*.*e*., the good solvents for lignin) at 298 and 473 K. Figure 3a,b presents *R*_g_ values in the presence of the Pd and C surfaces, respectively. Figure 4a shows that there is no significant solvent-mediated difference in lignin conformations in the presence of the Pd surface at 298 K. However, at 473 K, significantly higher *R*_g_ values are observed in the ethanol + water mixture compared to those of pure methanol and pure ethanol, indicating that lignin adopts more extended conformations in this solvent system in the presence of the Pd surface. This suggests that the ethanol + water mixture may provide a more favorable solvation environment for lignin at the high temperature associated with RCF reaction conditions, resulting in a more expanded structure that could promote depolymerization by exposing linkages to the catalyst. Notably, this solvent-mediated difference in lignin structure in the presence of Pd is absent in solution (Figure 4c) and is not anticipated based on the HSP analysis, highlighting the significant impact of the surface on the lignin structure at higher temperature.

**Figure 4:**
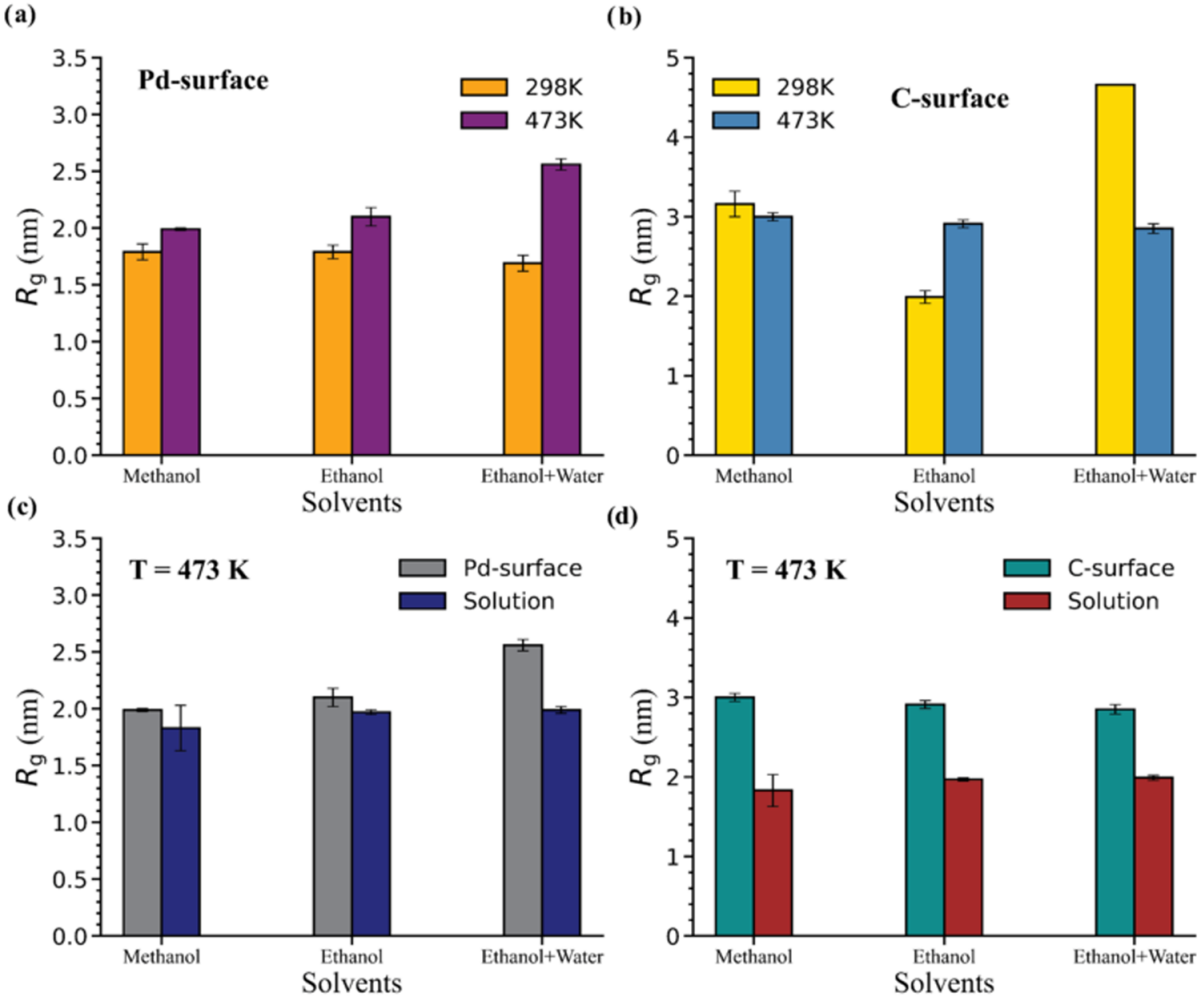
Average radii of gyration (*R*_g_) for the B1 lignin oligomer at 298 and 473 K in the presence of the (a) Pd and (b) C surfaces, along with comparisons of *R*_g_ values in solution to those in the presence of (c) Pd and (d) C surfaces at 473 K. Error bars represent the block-averaged standard error (method detailed in Section S3.1). Averages and error were calculated from the 200–500 ns interval of the production trajectory.

In the presence of the C surface (Figure 4b), all three solvent systems promote conformations more extended than those in the presence of the Pd surface at both temperatures. At 473 K, the similar *R*_g_ values across all of the solvents indicate that the solvent does not substantially affect lignin behavior, suggesting that lignin–surface interactions dominate over lignin-solvent interactions. Additionally, the higher *R*_g_ values in the presence of the C surface compared to those in solution (Figure 4d) indicate a substantial impact of the surface on the lignin structure. These behaviors are supported by the simulation snapshots in Figure S13, which show that lignin adsorbs similarly onto the C surface in all three solvents. Conversely, at 298 K lignin adopts a more extended conformation in ethanol + water, as indicated by higher *R*_g_ values compared to those in methanol and ethanol. This result indicates that at lower temperatures, the lignin-solvent interactions are more impactful than lignin interactions with the C surface, leading to a larger number of lignin segments being solvated away from the interfacial region, as further visualized in simulation snapshots in Figure S15. In contrast, in methanol and ethanol, lignin interacts more closely with the surface (also shown in Figure S15), which reduces lignin-solvent interactions and results in lower *R*_g_ values compared to that in ethanol + water. Overall, this analysis highlights that on average lignin–surface interactions promote more extended lignin conformations at the higher RCF temperature, especially on the C surface, resulting in similar lignin conformations across different solvents, whereas the presence of the Pd surface instead leads to solvent-dependent structural differences at high temperatures. The results also reveal variations in the lignin conformations obtained in the ethanol + water mixture that would not be expected based on HSPs or solvation in bulk solution, motivating further analysis in the sections below.

#### 3.2.2 Effects of Solvents on Lignin–Surface Contacts

The analysis of lignin conformations in bulk solution indicates that methanol, ethanol, and a binary mixture of ethanol + water can effectively solvate lignin, leading to extended conformations considered to be “catalytically competent”. These conformations expose the linkages to the surrounding environment, potentially facilitating subsequent catalytic reactions. Extended conformations are promoted in the presence of both the Pd surface (representative of the catalyst) and the C surface (representative of the support), suggesting that adsorption to the surface further exposes linkages to the catalyst. These findings motivate further quantitative analysis of how the choice of solvent impacts the adsorption of lignin onto the catalytic surface or support.

To explore how lignin interacts with the Pd and C surfaces, we computed the center-of-mass (COM) distance between each of the 28 subunits in the B1 lignin oligomer (19 S and 9 G subunits) and the top layer of the Pd/C surfaces at both temperatures. Figure 5 shows the number of subunits within a 1.0 nm cutoff distance from these surfaces at 298 and 473 K to quantify the extent of lignin–surface contact. These results demonstrate that there is a qualitative difference in the adsorption behavior of lignin across various solvents. At 298 K, methanol leads to significantly more subunits in contact with the surface, particularly on the C surface, indicating that lignin remains largely adsorbed to both surfaces throughout the 500 ns simulation trajectory. In ethanol, the number of subunits in contact with the surface fluctuates more substantially than in methanol, indicating that lignin transiently adsorbs and desorbs onto the Pd surface, while on the C surface, a smaller number of subunits than in methanol remain persistently adsorbed. In contrast, in ethanol + water, lignin prefers to stay in bulk solution and occasionally interacts with either surface. This behavior is further illustrated by simulation snapshots taken at various points along the trajectory (Figures S14 and S15). The comparison between methanol and ethanol is generally consistent with the HSPs, as the less favorable solvation by methanol is expected to promote surface interactions. However, the difference between ethanol and ethanol + water despite similar HSPs suggests variations in behavior in the presence of the solvent mixture, as further explored below.

**Figure 5:**
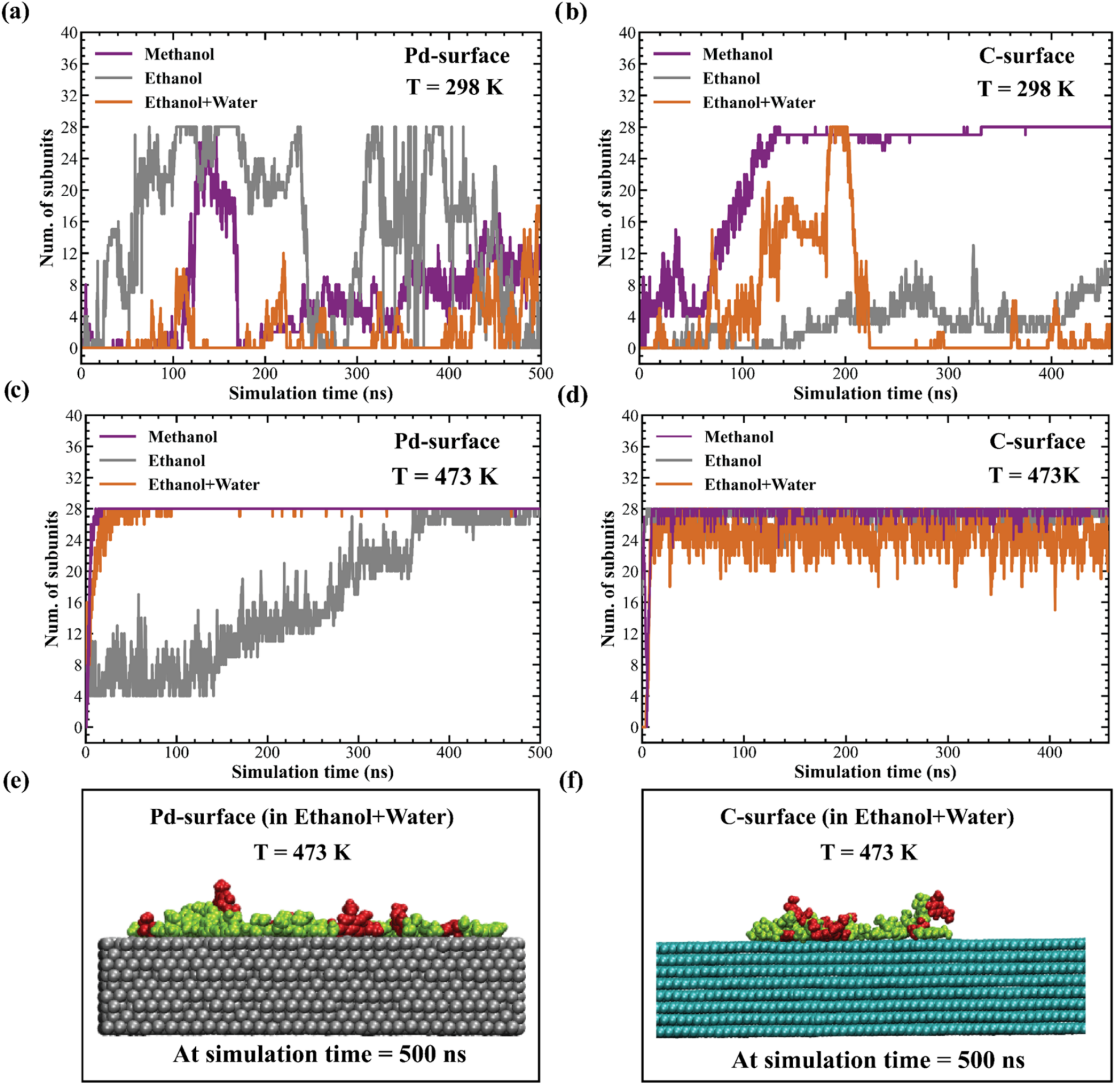
Number of subunits in the B1 oligomer with a center-of-mass distance within 1.0 nm of the top layer of the Pd/C surfaces as a function of the simulation time. Results are shown for methanol, ethanol, and ethanol + water systems at (a) 298 K on Pd surface, (b) 298 K on C surface, (c) 473 K on Pd surface, and (d) 473 K on C surface. Simulation snapshots illustrate the strong adsorption of lignin on the (e) Pd surface and (f) C surface at 473 K, with snapshots captured at the 500 ns simulation time point. Only the ethanol + water systems are shown as representative snapshots, while snapshots for the other solvent systems are shown in Figures S16 and S17. Solvent molecules are not shown for the sake of visual clarity.

In Figure 5c,d, at 473 K, the number of subunits in contact with the surface remains consistently high across all the solvents, indicating stronger and more stable adsorption onto both Pd and C surfaces at higher temperature. Figure 5e,f also shows representative snapshots illustrating the adsorbed configuration of lignin on Pd and C surfaces in ethanol + water at 473 K, while snapshots for the other solvent systems are shown in Figures S16 and S17. At this high temperature, lignin adopts an extended structure, as reflected by the higher *R*_g_ values observed at 473 K compared to those at 298 K (Figure 4a), that enables more surface contacts, thereby enhancing lignin adsorption onto both the Pd and C surfaces at 473 K. This observation is consistent with findings from previous studies which was reported that at higher temperatures the extended conformation of lignin facilitates greater surface contacts with a zeolite surface.^98^ The lower number of subunits in contact with the surface at 298 K compared with 473 K can be attributed to the lower *R*_g_ values of lignin at 298 K. The slightly compact structure of lignin at this temperature limits its ability to establish extensive contact with the surfaces. Hence, these findings indicate that temperature significantly influences lignin conformations and resulting interactions with the surface. A higher temperature promotes more favorable lignin–surface interactions, which could enhance catalytic efficiency.

#### 3.2.3 Lignin Adsorption Energies on Pd and C Surfaces

We further quantified solvent-mediated changes in the adsorption energies for the B1 lignin oligomer on Pd and C surfaces at 298 and 473 K using the approach outlined in Section 2.4. This approach computes separate contributions from lignin-solvent, lignin-solvent-surface, solvents-surface, and solvent–solvent interactions, providing a quantitative understanding of adsorption energetics at a reasonable computational cost compared to computationally expensive free energy calculations. In the following sections, we focus on adsorption at 473 K because Figure 5 suggests that all solvents promote strong adsorption at this temperature, and it is relevant to RCF reaction conditions; results for 298 K are shown in the SI. Tables S4 and S5 summarize the *E*_ads_ values, along with the individual energy components (*E*_1_, *E*_2_, *E*_3_ and *E*_4_) used in the adsorption energy calculations, for lignin adsorption on Pd and C surfaces in methanol, ethanol, and ethanol + water at both 298 and 473 K. The values are reported from two independent replica simulations, and the table also includes the average adsorption energy (*E*_ads,avg_) obtained from these replicas.

Figure 6 compares adsorption energies for lignin on Pd and C surfaces at 473 K across different solvents and includes averages over two independent replica simulations (*E*_ads,avg_). For the Pd surface, the average adsorption energy *E*_ads,avg_ is positive for methanol (877.5 kJ/mol) and ethanol (234.5 kJ/mol), while it is slightly negative (−115.5 kJ/mol) in the ethanol + water mixture. We note that while the magnitudes of these values appear large, the B1 oligomer contains 28 subunits (Table 1), such that the adsorption energies are between −4.1 and 31.3 kJ/mol per subunit or approximately −1.0 to 7.6 *k*_B_ *T* per subunit at 473 K, which are reasonable. For the C surface, *E*_ads,avg_ values vary among the solvents but are in general substantially larger, with negative values for ethanol (−1718.5 kJ/mol) and ethanol + water (−12,235.0 kJ/mol) but a positive value for methanol (588.0 kJ/mol). Positive *E*_ads,avg_ values indicate that adsorption is energetically unfavorable, whereas negative *E*_ads,avg_ values indicate that adsorption is energetically favorable. The increasingly negative *E*_ads,avg_ values for ethanol + water compared to ethanol and methanol on both surfaces and at both temperatures generally agree with the trend in *R*_g_ values, indicating that favorable adsorption energies drive extended conformations that maximize surface interactions.

**Figure 6:**
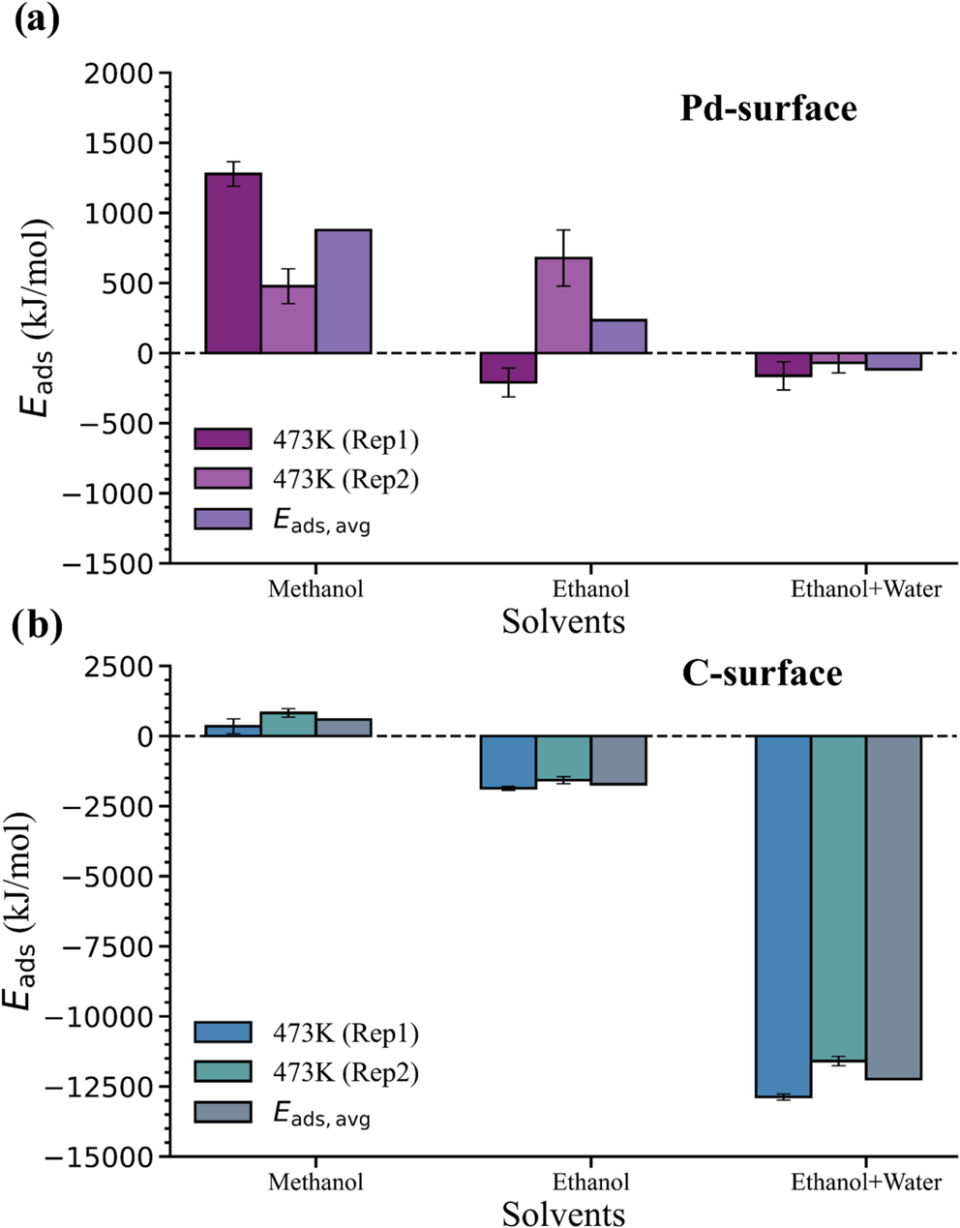
Adsorption energy, *E*_ads_, for lignin on (a) Pd and (b) C surfaces at 473 K for methanol, ethanol, and ethanol + water from two independent replicas. Standard errors in *E*_ads_ are computed using error propagation (detailed in Section S3.2), based on the standard errors of the individual system average energies (*E*_1_, *E*_2_, *E*_3_, and *E*_4_). *E*_ads,avg_ reports the average of the two replicas.

Surprisingly, several of the *E*_ads,avg_ values are positive, particularly in methanol, suggesting that lignin adsorption is not energetically favorable. In contrast, the lignin–surface contacts (Figure 5) indicated a strong, effectively irreversible adsorption of lignin on both Pd and C surfaces at 473 K in all solvents. To better understand these trends, we analyzed solvent-surface interactions, as they play a crucial role in determining the extent of lignin adsorption by influencing the competition between lignin and solvent for surface interactions. We calculated the difference in adsorption energy components (*E*_4_–*E*_3_) for both surfaces. *E*_4_ accounts for the solvent-surface, surface–surface, and solvent–solvent interactions, while *E*_3_ represents only the solvent–solvent interactions. To isolate the relevant contributions, we first subtracted the Lennard-Jones interaction energy for Pd–Pd and C–C from *E*_4_, ensuring that it includes only solvent–solvent and solvent-surface interactions. Finally, subtracting *E*_3_ from *E*_4_ isolates the solvent-surface interaction energy, providing insight into the solvent affinity for the surfaces. The (*E*_4_–*E*_3_) values for Pd and C surfaces in methanol, ethanol, and ethanol + water solvents at both temperatures are summarized in Tables S6 and S7, respectively. The corresponding trends are depicted in Figure S18.

For the Pd surface, (*E*_4_–*E*_3_) values are negative for all solvents at both temperatures, indicating a strong solvent affinity for the surface. Among the solvents, methanol exhibits the most negative (*E*_4_–*E*_3_) values, signifying that methanol strongly interacts with the Pd surface. This supports the earlier observation by showing that methanol competes with lignin for adsorption sites, leading to positive *E*_ads_. At the same time, methanol’s weak solvation strength, as also indicated by the HSPs, still allows lignin to remain near the surface. In contrast, for the C surface, *E*_4_–*E*_3_ values are positive for all solvents, indicating a weak solvent affinity for the surface. Ethanol and ethanol + water exhibit the highest positive *E*_4_–*E*_3_ values, suggesting the weakest solvent-surface interactions. As a result, ethanol and ethanol + water prefer to remain in the bulk, which is consistent with the nonpolar nature of the C surface and stronger solvent–solvent interactions in these systems.

Since adsorption is governed by the free energy of adsorption, the contrast between the adsorption energies (*E*_ads_) and persistent lignin–surface contacts (Figure 5) suggests that adsorption is entropically driven even for systems where the adsorption energy is unfavorable, particularly at higher temperatures, where adsorption is apparently stronger on both surfaces regardless of the solvent type. This analysis motivates additional investigation of potential entropic contributions to adsorption, as described in the following section.

### 3.3 Surface Solvation in the Presence and Absence of Adsorbed Lignin

To explore solvent-dependent entropic driving forces for lignin adsorption, we next investigated the structure of solvent molecules near the Pd and C surfaces. The role of interfacial solvent layers in mediating surface adsorption has been previously reported and is associated with the entropically unfavorable layering of solvent molecules due to interactions with a surface; displacing highly structured interfacial solvent molecules into the bulk thus is entropically favorable.^99–103^ We computed the number density of solvent molecules along the *z*-dimension of the simulation box for both surfaces at 298 and 473 K in the presence of lignin. The density profiles of all solvents for Pd at 473 K are shown in Figure 7, the profiles for C at 473 K are shown in Figure S19, and profiles at 298 K are shown in Figures S20 and S21. For the ethanol + water mixture, densities for ethanol and water are plotted separately to illustrate the relative affinities of the two mixture components for the surfaces.

**Figure 7:**
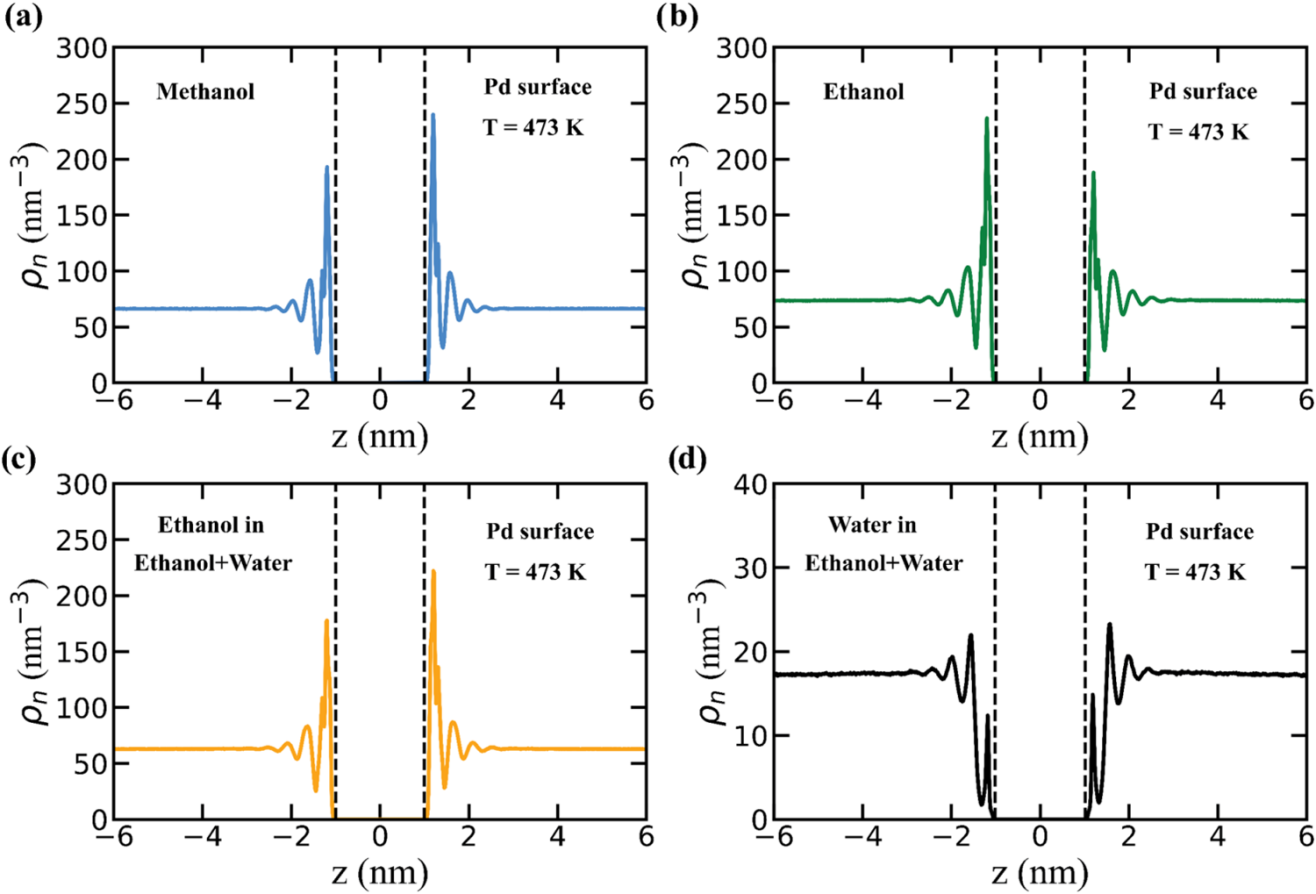
Number density (ρ_*n*_) profiles for solvents along the *z*-axis for pure methanol (a), pure ethanol (b), and ethanol (c) and water (d) in the ethanol + water mixture in the presence of the Pd surface at 473 K. Black dashed lines indicate the Pd surface, which presents two surfaces that interact with the solvent due to the periodic boundary conditions. The lower solvent density on one of the two surfaces is attributed to the displacement of solvent molecules by adsorbed lignin.

The density profiles in Figure 7 have well-structured solvation peaks near the Pd surface, indicating the presence of layers of solvent molecules near the surface. Similar results are obtained near the C surface, as shown in Figure S19. The structured layers of solvent molecules can be further visualized in simulation snapshots in Figure S22. However, as lignin adsorbs, some of the structured solvent molecules are displaced by the lignin. To quantify the extent of solvent displacement upon lignin adsorption, we integrated the first solvation peak on either side of the Pd surface to determine the number of solvent molecules in the presence and absence of lignin (since the lignin adsorbs to only one side of the surface). For the Pd surface in methanol, the number of solvent molecules near the surface decreases from 2641 in the absence of lignin to 2246 in its presence, indicating that 395 methanol molecules were displaced by lignin adsorption. Similarly, in ethanol, 833 molecules were displaced. In ethanol + water, lignin displaces 386 ethanol molecules and 12 water molecules, indicating that water is already excluded from the surface, whereas ethanol is preferentially retained near the surface. For the C surface, a similar trend is obtained: 636 methanol molecules were displaced, 732 ethanol molecules were displaced, and in ethanol + water mixture, 136 ethanol molecules and 131 water molecules were displaced. The C surface shows lower ethanol displacement and more water displacement, indicating a structured solvent environment near the surface with lower entropy of mixing.

The lignin–surface contact calculations (Figure 5) suggest rapid, irreversible lignin adsorption onto both Pd and C surfaces at 473 K even though some solvents (such as methanol) exhibit positive adsorption energies. This solvent analysis suggests that strong adsorption is driven by the displacement of structured solvent molecules, leading to a significant entropy gain due to increased rotational and translational freedom of the liberated solvent molecules. The preferential displacement of ethanol molecules in the ethanol + water mixture is particularly favorable not only due to the displacement of structured solvent molecules from the surface but also due to the entropy of mixing as displaced ethanol molecules reenter into the bulk solvent mixture from the surface region which is enriched in ethanol. This additional contribution from mixing explains the deviation in behavior for ethanol + water mixtures compared to ethanol despite their similar solvation behavior according to HSP analysis. These entropic factors are particularly pronounced at high RCF reaction temperatures because of the increased contribution of entropy to free energies of adsorption at higher temperatures. This behavior thus highlights the role of solvent molecules in providing an entropic driving force for adsorption to complement the adsorption energies.

It is important to note that all surface-related analyses presented in Sections 3.2 and 3.3 were performed using the B1 oligomer, because its structural characteristics best represent experimental reports for poplar lignin. The other oligomers (B2–B4, M5) differ in S/G ratio, molecular weight, and interunit linkages and could therefore possess different affinities for model surfaces (as further discussed below). However, we do not expect the observed solvent-dependent trends to change significantly because all five lignin oligomers exhibit similar behavior in bulk solution, as shown by comparable *R*_g_ and SASA values in the various organic solvents (Table 2). Although future work is needed to systematically assess the effect of structural heterogeneity on surface interactions, the current results provide valuable insights into how different solvent environments govern lignin adsorption behavior under RCF conditions.

### 3.4 Free Energy of Monomer Adsorption

The oligomer adsorption energy calculations and associated analysis of the solvent structure indicate that the adsorption behavior depends upon the choice of solvent and is entropically driven, particularly at the high RCF reaction temperature. The observation of persistent lignin–surface contacts in Figure 5 also indicates strong, irreversible adsorption of oligomers, which we attribute to entropic driving forces. However, the adsorption energy calculations themselves do not directly report on this free energy. To more directly probe adsorption thermodynamics, we computed the potential of mean force (PMF) for a single lignin monomer—either syringyl (S) or guaiacyl (G)—as a function of the *z*-component of the distance (*d*_*z*_) from the Pd surface in methanol, ethanol, and ethanol + water at 473 K. These calculations are intended to complement and validate the oligomer adsorption energy calculations for the systems most relevant to RCF. Specifically, we sought to determine whether (1) monomer adsorption free energies are highly favorable, as suggested by the persistent lignin–surface contacts in Figure 5; (2) monomer adsorption energies are either weakly favorable or unfavorable, in agreement with the oligomer adsorption energy calculations in Figure 6; and (3) adsorption is driven entropically due to the release of solvent molecules as suggested by Figure 7. PMF convergence for a representative system is shown in Figure S23.

As shown in Figure 8, the PMF profiles exhibit well-defined free energy minima at *d*_*z*_ ≈ 0.5 nm for both monomers, corresponding to the adsorbed state in which the monomer resides in direct contact with the Pd surface. The PMF profiles plateau between *d*_*z*_ ≈ 1.4–2.0 nm, indicating a fully desorbed state. The adsorption free energy (Δ*G*_ads_) was calculated as the difference between the global minimum (adsorbed state) and the plateau region (desorbed state) of each profile. Δ*G*_ads_ values range from −80 to −85 kJ/mol for the S monomer and from −65 to −78 kJ/mol for the G monomer depending upon the solvent (Table 3). These values correspond to approximately −20.3 to −21.4 *k*_*B*_ *T* for S and −16.5 to −19.8 *k*_*B*_ *T* for G at 473 K, indicating highly favorable adsorption and a negligible probability of spontaneous desorption. This strong thermodynamic preference for adsorption supports the results in Figure 5, which show that the number of oligomer subunits in contact with the surface remains consistently high across all solvent systems at 473 K.

**Figure 8:**
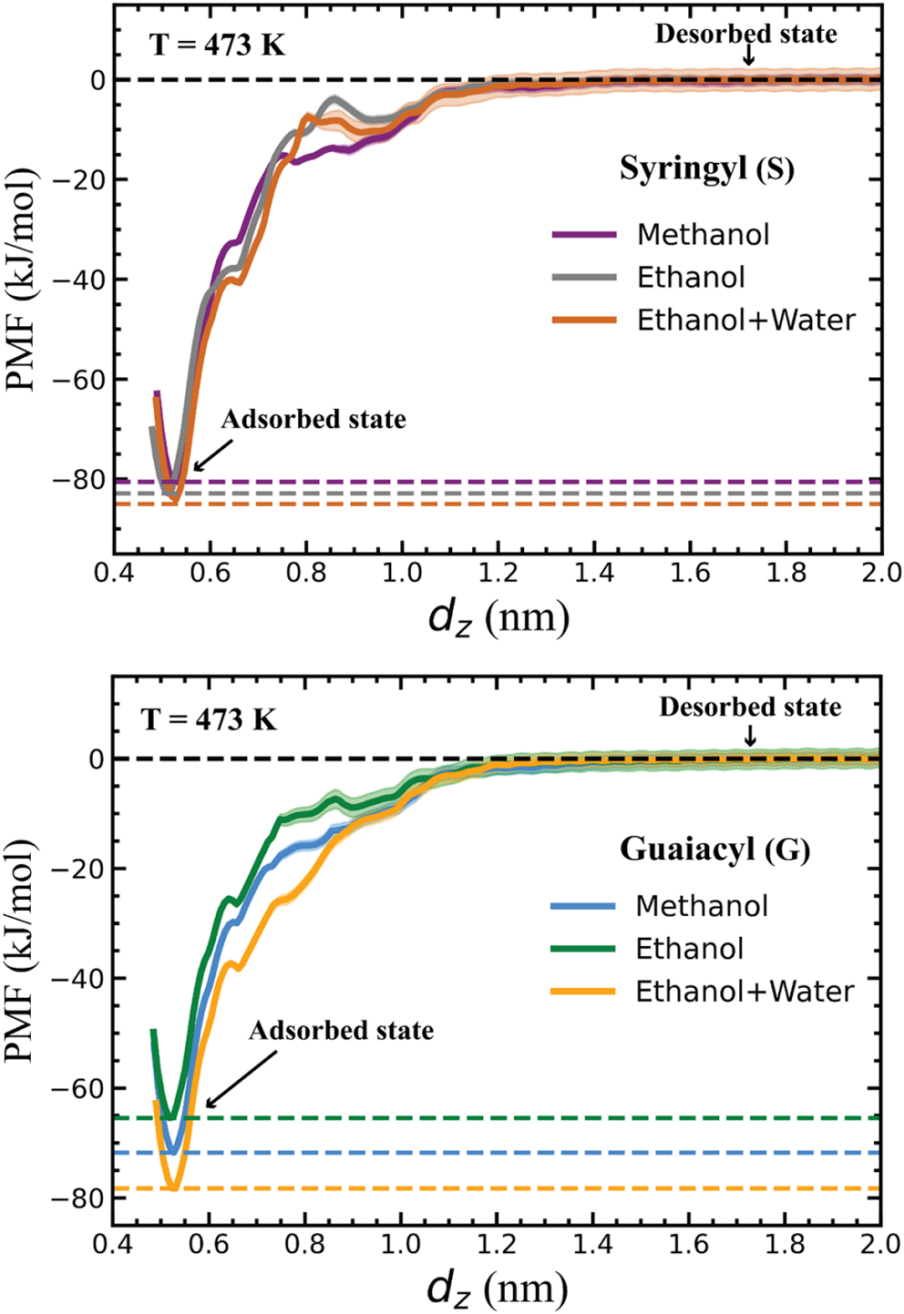
Potentials of mean force (PMFs) as a function of the *z*-component of the distance between the center-of-mass (COM) of the monomer and COM of the top layer of the Pd surface, *d*_*z*_. The PMF profiles represent the adsorption of syringyl (S) and guaiacyl (G) monomers onto the Pd surface in different solvents (methanol, ethanol, and ethanol + water) at 473 K. The shaded area indicates the standard deviation computed by bootstrapping.

**Table 3:** Adsorption Free Energy (Δ*G*_ads_), Enthalpy (Δ*H*), Energy (Δ*E*), and Entropy (−*T*Δ*S*) for Syringyl and Guaiacyl Monomers on the Pd Surface at 473 K^a^

**syringyl (S) monomer**			
(kJ/mol)	methanol	ethanol	ethanol + water
Δ*G*_ads_	–80.6	–82.9	–85.0
Δ*H*	18.5	1.6	–1.7
Δ*E*	9.5	0.1	–0.7
–*T*Δ*S*	–56.1	–41.8	–44.9

**guaiacyl (G) monomer**			
(kJ/mol)	methanol	ethanol	ethanol + water
Δ*G*_ads_	–71.7	–65.4	–78.3
Δ*H*	–23.5	–4.5	–11.2
Δ*E*	–21.4	–5.7	–11.4
–*T*Δ*S*	–19.9	–27.3	–29.4

a Δ*G*_ads_ values were obtained from the PMFs, Δ*H* and −*T*Δ*S* were obtained from the decomposition of the PMFs, and Δ*E* corresponds to the average internal energy difference between the adsorbed and desorbed states.

We next decomposed the adsorption free energy into enthalpic and entropic components by subtracting the ensemble-averaged enthalpy from the PMF-derived free energy and then further computed the average internal energy. The adsorption enthalpy (Δ*H*), entropy (reported as −*T*Δ*S*), and energy (Δ*E*) were then computed as the difference between values for the adsorbed state (defined by averaging over a range of 0.5 nm < *d*_*z*_ < 0.7 nm) and the desorbed state (defined by averaging over 1.75 nm < *d*_*z*_ < 1.95 nm). Table 3 presents Δ*G*_ads_, along with the corresponding Δ*H*, Δ*E*, and −*T*Δ*S* components for both S and G monomers across all solvent systems. The results consistently demonstrate that adsorption is primarily driven by entropy rather than energy. For instance, in the case of the S monomer in methanol, Δ*E* = 9.5 kJ mol^–1^ while −*T*Δ*S* = −56.1 kJ mol^–1^, with an overall adsorption free energy Δ*G*_ads_ = −80.6 kJ mol^–1^. Similar trends are observed for the S monomer in ethanol and ethanol + water, where entropic contributions dominate (−41.8 kJ mol^–1^ for ethanol and −44.9 kJ mol^–1^ for ethanol + water) despite small or unfavorable energetic components. While these calculations cannot be quantitatively compared to the results for the lignin oligomer because the monomers lack the connectivity of the complete lignin oligomer, they are in strong qualitative agreement with the adsorption energy analysis; namely, they illustrate that adsorption energies can be positive and yet adsorption can be highly favorable due to solvent-mediated entropic driving forces. For the S monomer, the rank order of monomer adsorption energies (Δ*E*) also agrees with the lignin oligomer adsorption energies (Figure 6), for which methanol led to a large positive value, ethanol was closer to zero, and ethanol + water was slightly negative. In contrast, for the G monomer, both Δ*E* and −*T*Δ*S* are negative, with Δ*H* ranging from −21.4 to −5.7 kJ mol^–1^ and −*T*Δ*S* ranging from −19.9 to −29.4 kJ mol^–1^ across the solvents. This indicates that for the G monomer, both energetic and entropic contributions favor adsorption, although entropy is on average a more significant role. Since the B1 lignin oligomer has a 2.1 S/G ratio, the larger contribution of S subunits likely leads to the positive adsorption energies.

Finally, to quantify the role of released solvent molecules on the monomer adsorption energy, we calculated the number of solvent molecules displaced during monomer adsorption by integrating the first solvation peak on either side of the Pd surface for the windows in which the monomer was adsorbed. The results are summarized in Table S8, which reports the number of solvent molecules near the surface (within 0.6 nm) at 473 K, both in the presence and absence of S and G monomers. The difference reflects the number of displaced solvent molecules, providing a direct link to the entropic gain observed in the PMF decomposition and similar calculations for the lignin oligomers (Figure 7). Table S8 indicates that the number of solvent molecules displaced upon adsorption ranges from 12 to 24, which is reasonable given the bulky and planar structures of the S and G monomers and consistent with the large number of solvent molecules displaced by oligomer adsorption. This displacement correlates well with the observed entropic contributions (Table 3), further supporting the interpretation that the entropy gained from solvent displacement is a dominant factor in the adsorption process. Together, the monomer adsorption free energy calculations provide additional support for the key findings of the adsorption energy and solvent analysis for the lignin oligomers, while also pointing to differences between monomers that highlight the potential impact of structural heterogeneity on surface adsorption.

## 4 Conclusions

In this study, we utilized atomistic MD simulations to investigate the solvation behavior of oligomeric lignin compounds in various organic solvents, such as methanol, ethanol, a binary mixture of ethanol and water (85:15, v:v), and water at both room temperature (298 K) and a typical RCF reaction temperature (473 K). The choice of solvents and simulation conditions were guided by experimentally reported data on poplar lignin.^50^ To explore the conformational changes of the lignin in different solvents, we analyzed structural features such as the radius of gyration (*R*_g_) and solvent-accessible surface area (SASA). At 298 K, the *R*_g_ and SASA analyses for the lignin oligomers show similar solvation behavior in methanol, ethanol, and the ethanol + water mixture, indicating that all of these solvent systems promote extended lignin conformations suitable for catalytic conversion. We do not observe any significant temperature-dependent changes in lignin structure in bulk solution. Conversely, introducing model Pd and C surfaces revealed distinct solvent-mediated effects on lignin conformations, especially at a higher temperature. In the presence of Pd surface at 473 K, lignin adopted a more extended structure in the ethanol + water solvent mixture compared to methanol and ethanol. This solvent-mediated structural difference, which is absent in bulk solution, highlights the effect of surface interactions. On the C surface at 473 K, *R*_g_ values of lignin are similar across all solvents, indicating that lignin–surface interactions dominated over lignin-solvent interactions, resulting in uniform conformations in all solvent systems. We further computed solvent-mediated adsorption energies to reveal quantitative differences in the adsorption behavior. Our results show that for both surfaces, the ethanol + water mixture leads to the strongest adsorption, followed by pure ethanol. Methanol, surprisingly, leads to positive adsorption energies despite observations of strong, irreversible adsorption on both surfaces at 473 K. Analysis of interfacial solvent structure and individual monomer adsorption free energies shows that a substantial number of structured solvent molecules are liberated upon lignin adsorption, indicating that entropic driving forces promote adsorption even if adsorption energies are weak or positive.

The findings of this study provide molecular insights into how the solvent choice influences lignin adsorption on Pd and C surfaces. Our results suggest that ethanol and ethanol + water mixtures may be preferred over methanol for RCF due to their ability to facilitate lignin adsorption onto catalytic surfaces while avoiding excessively strong interactions that could hinder product desorption. In particular, the extended lignin conformations observed in ethanol and ethanol + water at 473 K suggest that these solvents provide a balance between the lignin solvation and the lignin–surface interactions. Moreover, the support material plays a crucial role in lignin–surface interactions, which can significantly impact the RCF efficiency. Our simulations show that the carbon surface promotes strong lignin adsorption and extended conformations across all solvents. While carbon supports are commonly used for structural stability, this degree of strong lignin adsorption could be unfavorable because they compete against interactions with the catalyst. These findings highlight the need to consider both solvent effects and support materials while designing RCF processes. Future studies will focus on understanding product desorption from the surfaces to further refine solvent and support choices, ensuring optimal conditions for lignin depolymerization.

## Supplementary Material



## Notes

Additional simulation methodological details, additional simulation results and snapshots, data supporting method convergence, and tables showing simulation results (PDF)
