# Supplementary material for: Effect of Solvents on Lignin–Surface Interactions via Molecular Dynamics Simulations
Source: J Phys Chem B. 2025 Aug 27;129(36):9175–89. doi: 10.1021/acs.jpcb.5c02943 (PMC12503381; doi:10.1021/acs.jpcb.5c02943)
Supplement: Supplementary file 1 [file jp5c02943_si_001.pdf]

Supporting Information

Effect of Solvents on Lignin-Surface Interactions via Molecular Dynamics Simulations

Juriti Rajbangshi[1, 2], Canan Sener[2,4], and Reid C. Van Lehn[1,2,3,*]

[1]Department of Chemical and Biological Engineering, University of Wisconsin−Madison, Madison, Wisconsin 53706, United States

[2]DOE Great Lakes Bioenergy Research Center, University of Wisconsin−Madison

[3]Department of Chemistry, University of Wisconsin−Madison, Madison, Wisconsin 53706, United States

[4]Wisconsin Energy Institute, University of Wisconsin-Madison, Madison, Wisconsin 53726, United States

*to whom correspondence should be addressed; vanlehn@wisc.edu

Section S1: Simulation system preparation

We modeled Pd and C surfaces by utilizing the CHARMM-GUI Nanomaterial Modeler[1,2] with parameters for Pd from the Interface Force Field (IFF)[3]. We built a 3D periodic slab as a model of the Pd(111) surface with an area of $9.07698 \times 8.09931\ \text{nm}^2$, a thickness of 2.02131 nm and a total height of 13.02131 nm, and a C-surface with an area of $9.37460 \times 8.11870\ \text{nm}^2$, thickness of 2.67840 nm and total height of 13.67840 nm. For the C-surface, we used a slab of graphite from the Carbonaceous Nanomaterials section of the Nanomaterial Modeler. We selected 11.0 nm as the size of the z-axis of the simulation box to ensure the lignin oligomer only interacted with one side of each 3D slab. The lignin oligomer was randomly inserted into the simulation box by using the *gmx insert-molecules* tool, and then solvated using the *gmx solvate* tool. During solvation, the solvent configurations were derived from an equilibrated simulation of bulk solvent. Figure S1 shows a schematic representation of two simulation boxes, one containing lignin on Pd-surface in methanol and the other containing with lignin on C-surface in methanol. A similar approach was used to set up systems for the monomer adsorption free energy calculations. For these systems, we built a Pd(111) surface with an area of $4.12 \times 3.33\ \text{nm}^2$, a thickness of 1.34 nm, and a total height of 9.23 nm, with systems containing 1292 methanol molecules, 889 ethanol molecules, or 844 ethanol and 483 water molecules depending on the chosen solvent.

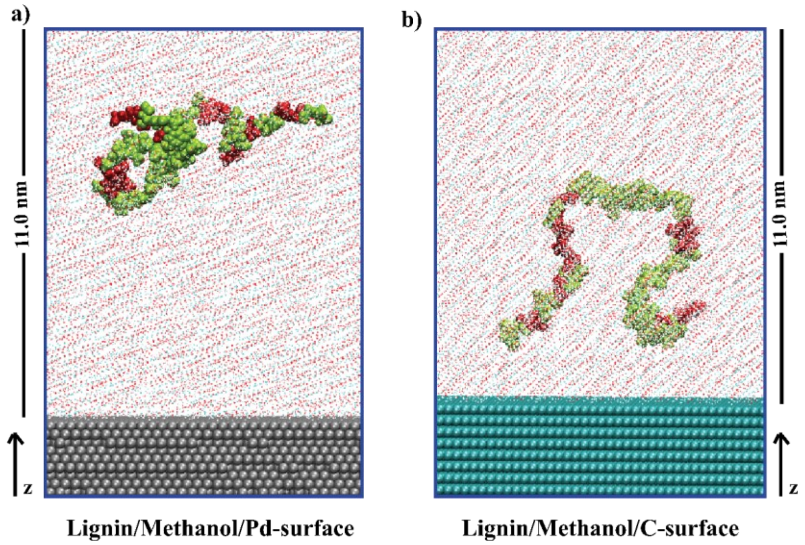

Figure S1. Initial setup of the surface simulation. Periodic simulation boxes of lignin in methanol in the presence of the (a) Pd-surface and (b) C-surface.

Table S1. Simulation systems for adsorption energy calculations in the presence of the Pd surface.

Solvent	Simulation System	Box Dimensions nm^3	No. of solvent molecules
Methanol	Lignin + Solvent + Surface	$(9.07 \times 8.09 \times 13.02)$	11395
	Lignin + Solvent	$(9.07 \times 8.09 \times 11.0)$	11555
	Bulk Solvent	$(9.07 \times 8.09 \times 11.0)$	11726
	Solvent +Surface	$(9.07 \times 8.09 \times 13.02)$	11557
Ethanol	Lignin + Solvent + Surface	$(9.07 \times 8.09 \times 13.02)$	7718
	Lignin + Solvent	$(9.07 \times 8.09 \times 11.0)$	7881
	Bulk Solvent	$(9.07 \times 8.09 \times 11.0)$	8026
	Solvent +Surface	$(9.07 \times 8.09 \times 13.02)$	7845
Ethanol+Water[a]	Lignin + Solvent + Surface	$(9.07 \times 8.09 \times 13.02)$	7090:4057
	Lignin + Solvent	$(9.07 \times 8.09 \times 11.0)$	7090:4057
	Bulk Solvent	$(9.07 \times 8.09 \times 11.0)$	7090:4057
	Solvent +Surface	$(9.07 \times 8.09 \times 13.02)$	7090:4057

[a]Volume fraction of Ethanol+Water = 85:15, number of ethanol molecules = 7090, number of water molecules = 4057.

Table S2. Simulation systems for adsorption energy calculations in the presence of the C surface.

Solvent	Simulation Systems	Box Dimensions nm^3	No. of solvent molecules
Methanol	Lignin + Solvent + Surface	$(9.37 \times 8.12 \times 13.68)$	11917
	Lignin + Solvent	$(9.37 \times 8.12 \times 11.0)$	12025
	Bulk Solvent	$(9.37 \times 8.12 \times 11.0)$	12187
	Solvent +Surface	$(9.37 \times 8.12 \times 13.68)$	12078
Ethanol	Lignin + Solvent + Surface	$(9.37 \times 8.12 \times 13.68)$	8163
	Lignin + Solvent	$(9.37 \times 8.12 \times 11.0)$	8281
	Bulk Solvent	$(9.37 \times 8.12 \times 11.0)$	8412
	Solvent +Surface	$(9.37 \times 8.12 \times 13.68)$	8299
Ethanol+Water[a]	Lignin + Solvent + Surface	$(9.37 \times 8.12 \times 13.68)$	7340:4199
	Lignin + Solvent	$(9.37 \times 8.12 \times 11.0)$	7340:4199
	Bulk Solvent	$(9.37 \times 8.12 \times 11.0$	7340:4199
	Solvent +Surface	$(9.37 \times 8.12 \times 13.68)$	7340:4199

[a]Volume fraction of ethanol+water = 85:15, number of ethanol molecules = 7340, number of water molecules = 4199.

Section S2: Calculation of Adsorption Energy

We computed energies for the adsorption of lignin onto the Pd and C surfaces by utilizing an approach previously developed by Heinz *et al.*[4,5]. This approach involves simulating four independent systems, containing: (1) lignin, surface, and solvent; (2) lignin and solvent; (3) solvent only; and (4) surface and solvent. The four simulation systems were designed to separate various energy contributions (see Figure S3) by modeling subsets of system components as follows:

(1) **Lignin + surface + solvent system:** Lignin adsorbed onto the surface in the presence of solvent. This system's ensemble-average energy is denoted as E_1.

(2) **Lignin + solvent system:** Lignin in bulk solution without the surface. This system's ensemble-average energy is denoted as E_2.

(3) **Solvent system:** Solvent only without lignin or the surface. This system's ensemble-average energy is denoted as E_3

(4) **Solvent + surface system:** Solvent interacting with the surface without lignin. This system's ensemble-average energy is denoted as E_4.

The adsorption energy (E_{ads}) is calculated from the ensemble-average energies for each of these four systems using the equation,

$$E_{ads} = E_1 - E_2 + E_3 - E_4 \tag{S1}$$

This approach accounts for the interactions between lignin and the surface, lignin and the solvent, solvent and the surface, and solvent with itself, as illustrated in Figure S3. Eq. S1 systematically subtracts interaction terms to ensure that the adsorption energy (E_{ads}) reflects only the energy contribution from the interaction between the lignin oligomer and the surface.

All four simulations were performed by considering equal molecular volumes for each system, preventing artifacts that could arise from variations in system size or solvent density. By keeping the cross-sectional area of the simulation box constant, the box height (z) is adjusted appropriately to accommodate different components (lignin, surface, solvent). This approach ensures that all simulation boxes maintain the same total solvent volume. The box dimension and number of molecules used for both Pd and C surfaces are provided in Tables S1 and S2, which show similar system sizes across the four simulations.

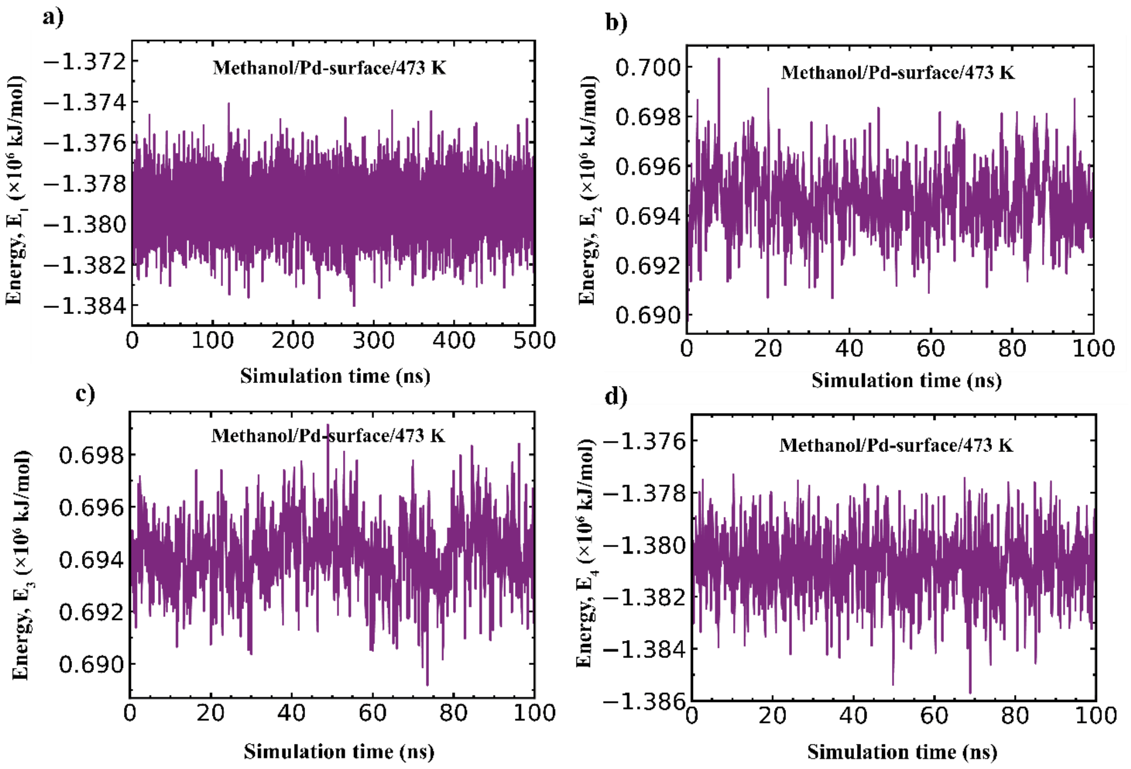

Figure S2. Example system energies as a function of simulation time for the calculation of the adsorption energy on Pd in methanol at 473 K. (a) E_1, average energy of the lignin-solvent-Pd system, (b) E_2, average energy of the lignin-solvent system, (c) E_3, average energy of the pure solvent system, (d) E_4, average energy of the solvent-Pd surface.

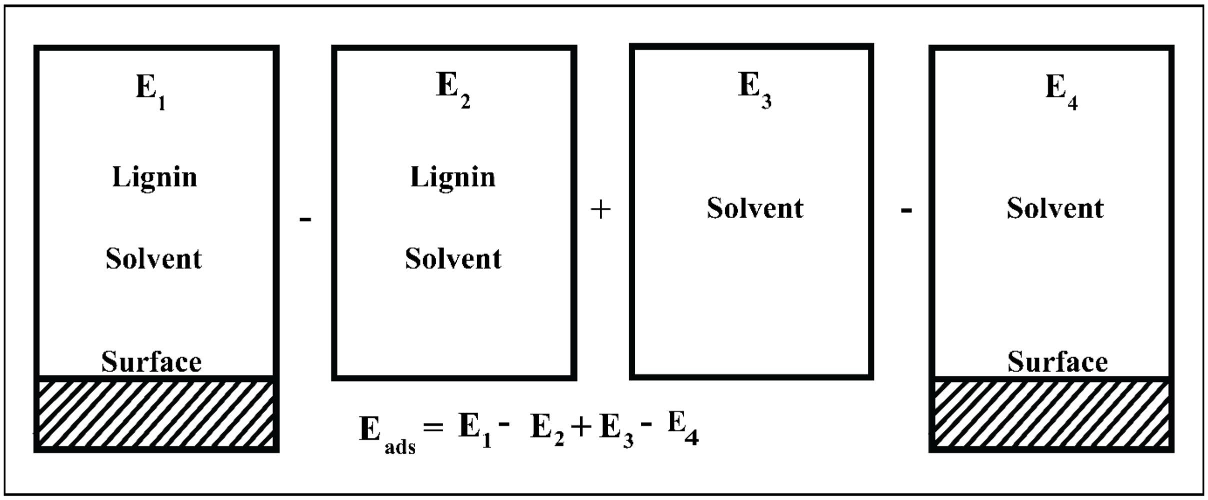

Figure S3. Computation of adsorption energies. Adsorption energies computed by employing this approach involves contributions from lignin-solvent, lignin-surface, solvent-surface, and solvent-solvent interactions. Here, illustrations are shown in 2D for simplicity.

Section S3: Uncertainty Quantification

Block averaging was used to quantity standard error for the R_g and SASA calculations and adsorption energies. For adsorption energies, standard errors for each individual energy $E_{i(i=1,2,3,4)}$ were obtained using block averaging over a number of segments of the total simulation trajectory. The standard error of the adsorption energy, (E_{ads}) was determined through error propagation based on the standard errors of the individual system energies (E_1, E_2, E_3 and E_4). Adsorption energies are reported from two independent replicas of the entire simulation workflow (*i.e.*, replicas of all four systems), each initialized with different starting configurations.

Section S3.1 Block averaging

The block averaging method can be summarized as follows: a simulation trajectory containing $N = M \times n$ configurations is divided into M segments (or "blocks"), where n represents the number of configurations in each block.[6] The average energy is computed for each block, resulting in M values for $\langle E_i \rangle$ with $i = 1, 2, 3, \ldots, M$. For each value of n, the standard deviation among the block averages, denoted as σ_n, is used to calculate a running estimate of the overall standard error, expressed as,

$$\text{Block standard error, BSE } (E; n) = \frac{\sigma_n}{\sqrt{M}}$$

This is the standard error based on blocks of length n. Then, a convergence plot as generated to visualize the relationship between BSE and n. By analyzing this plot, the converged block length is identified, and the final standard error is calculated by using converged block length. For the R_g and SASA calculations, we used n = 10 blocks and for the adsorption energy calculations we used n = 100 blocks.

Section S3.2 Error propagation

To compute the standard deviation of the adsorption energy, E_{ads} we apply the principles of error propagation. The variance and standard error for the adsorption energy, $E_{ads} = E_1 - E_2 + E_3 - E_4$ can be obtained as

$$\sigma_{E_{ads}}^2 = \sigma_{E_1}^2 + \sigma_{E_2}^2 + \sigma_{E_3}^2 + \sigma_{E_4}^2$$

$$\sigma_{E_{ads}} = \sqrt{\sigma_{E_1}^2 + \sigma_{E_2}^2 + \sigma_{E_3}^2 + \sigma_{E_4}^2}$$

Here, the covariance is assumed to be zero, as the uncertainties in E_1, E_2, E_3 and E_4 are independent. The standard errors σ_{E_1}, σ_{E_2}, σ_{E_3} and σ_{E_4} are obtained through block averaging over multiple segments of the total simulation trajectory, as detailed in section S3.1.

Section S4: Additional Simulation Results

S4.1 Radius of gyration analysis

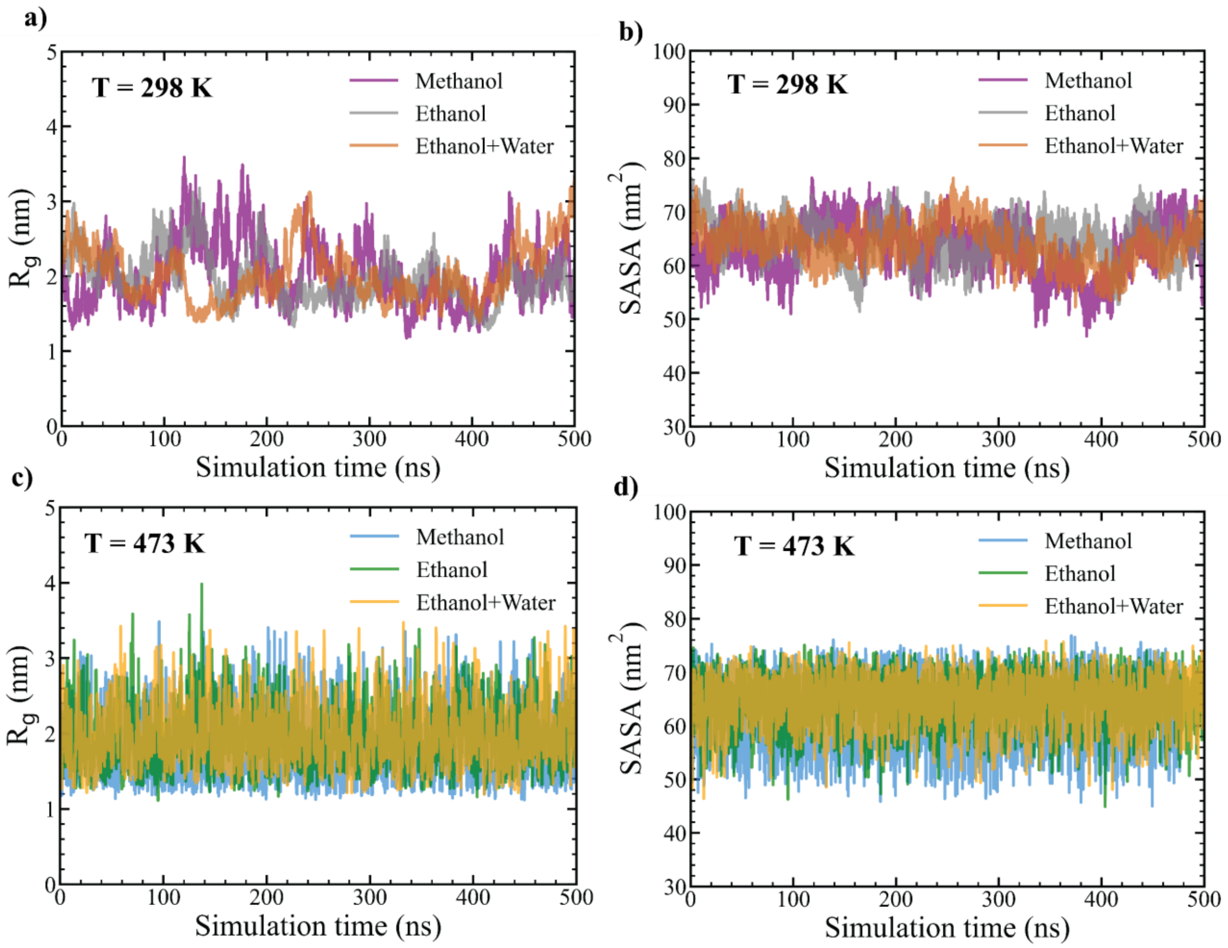

Figure S4. Radii of gyration (R_g) and solvent-accessible surface area (SASA) as a function of time for the B1 lignin oligomer. (a) and (b) show R_g at 298 K and 473 K, respectively, while (c) and (d) show the SASA at 298 K and 473 K, respectively. Average values and uncertainty for these quantities were computed using the time interval from 200-500 ns.

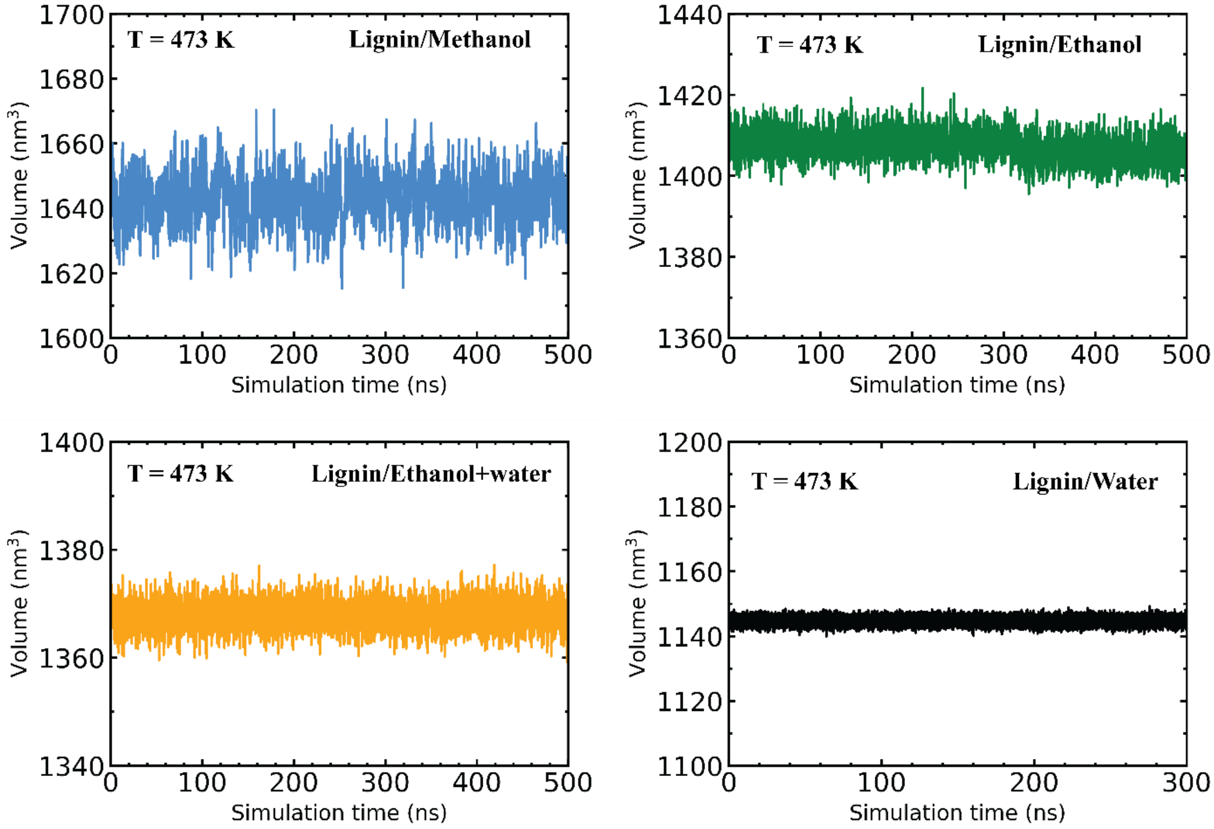

Figure S5. Simulation volume as a function of time during the NPT production trajectory for the B1 lignin oligomer in methanol, ethanol, ethanol+water and water at 473 K.

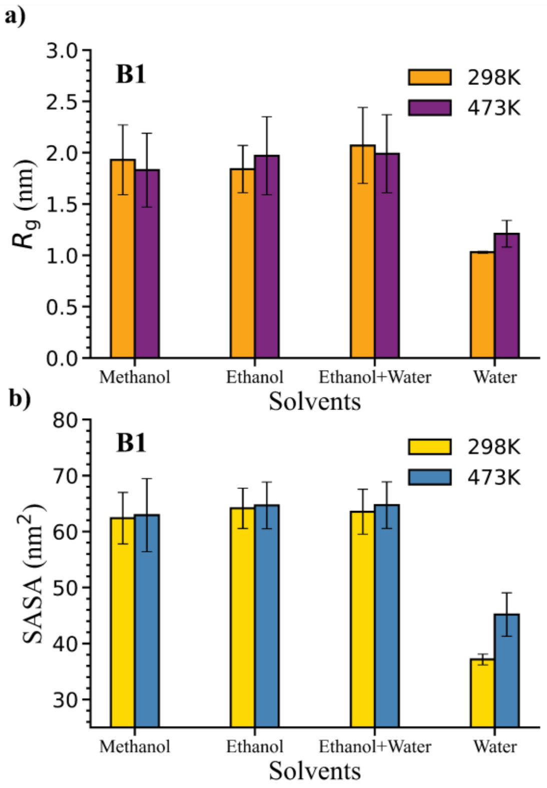

Figure S6. Analysis of lignin structures in bulk solution. Two simulation quantities are computed for the B1 lignin oligomer at 298 K and 473 K: a) the average radius of gyration (R_g) and b) the solvent-accessible surface area (SASA). Average R_g and SASA values for other oligomers at 298 K are provided in Table 2. Error bars represent the standard deviation over the production trajectory as a measure of fluctuations. Averages and error were calculated from the 200-500 ns interval of the production trajectory.

S4.2 Simulation snapshots in various solvent environments

Figure S7 shows simulation snapshots of the B1 lignin oligomer in different solvent environments. This figure illustrates extended conformation in organic solvents (methanol/ethanol/ethanol+water) and a collapsed structure in pure water. The R_g and SASA analyses in the main text further quantify these conformational changes of lignin.

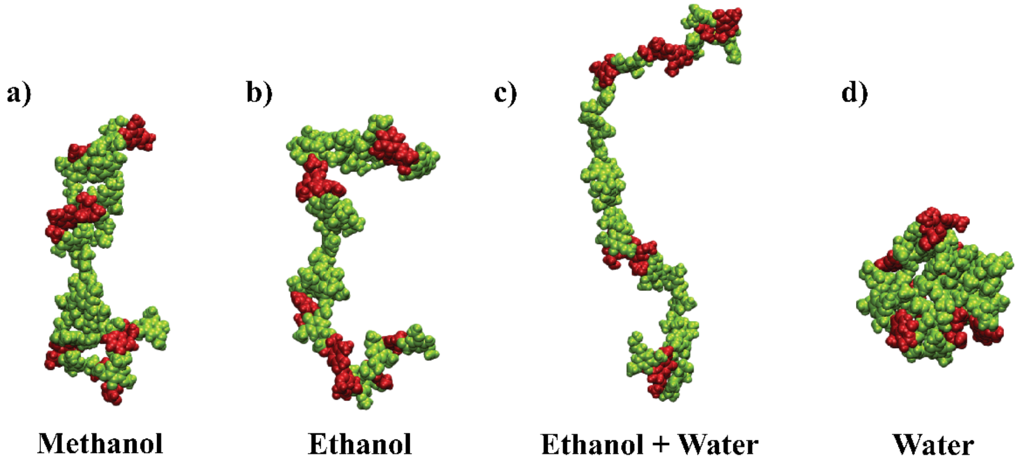

Figure S7. Simulation snapshots of B1 oligomer in methanol, ethanol, ethanol+water and water at 298 K. Here, green represents SYR and red represents GUAI subunits. Solvent molecules are not shown for visual clarity.

Hansen Solubility Parameters (HSPs)

Hansen solubility parameters (HSPs) can be used to screen and predict solvents that are favorable for lignin solvation. The three HSPs quantify the strength of dispersion interactions (δ_D), dipole-dipole interactions (δ_P), and hydrogen bonding interactions (δ_H). HSPs for a wide range of pure solvents have been already tabulated based on empirical measurements to achieve self-consistent values[7]. HSPs for methanol, ethanol, ethanol+water and water are provided in Table S3. HSPs for the ethanol+water (85:15 v:v) mixture are calculated using the volume-fraction-weighted average of individual solvent parameters, which can be expressed as $(\delta_D^m, \delta_P^m, \delta_H^m) = \left(\frac{\sum_i \phi_i \delta_{D,i}}{\sum_i \phi_i}, \frac{\sum_i \phi_i \delta_{P,i}}{\sum_i \phi_i}, \frac{\sum_i \phi_i \delta_{H,i}}{\sum_i \phi_i}\right)$ where ϕ_i is the volume fraction of solvent *i* in the mixture.

It is well-known that the solubility of a polymer in a solvent can be assessed using the HSPs by defining a solubility sphere in HSP space, centered on the HSPs of the polymer, such that solvents promoting polymer dissolution fall within this sphere (as shown in Figure S8). The radius of the solubility sphere (R_0) depends on the polymer's solubility in the reference solvent systems. Solvents within the sphere can be identified by calculating the distance (R_a) between the polymer (*poly.)* and solvent (*solv.*) in $\delta_D - \delta_P - \delta_H$ space, as shown in equation S1.

$$R_a^2 = 4(\delta_D^{solv.} - \delta_D^{poly.})^2 + (\delta_P^{solv.} - \delta_P^{poly.})^2 + (\delta_H^{solv.} - \delta_H^{poly.})^2 \quad \text{(S2)}$$

The solubility of a polymer can then be characterized by computing the Relative Energy Difference (RED), which is defined as the ratio of R_a to the interaction radius of the polymer (R_0),

$$\text{RED} = R_a/R_0 \quad \text{(S3)}$$

A RED value greater than unity suggests that the polymer will not dissolve in a solvent, whereas a RED value less than unity suggests that it will dissolve in the solvent. RED calculations have been extensively used to screen and predict solvents that are favorable for lignin solvation[8–10] although HSPs for lignin can vary depending upon the feedstock. We calculated RED values for all the solvents by using HSPs for lignin reported by Curvalo et al.[10] As summarized in Table S3, the resulting RED values indicates that the organic solvents are expected to act as good solvents

for lignin, whereas water behaves as a poor solvent. Note that although the RED value for methanol (1.03) slightly exceeds unity, it is still considered as good solvent, given that we used representative lignin HSPs that may vary slightly for different lignin S/G ratios.

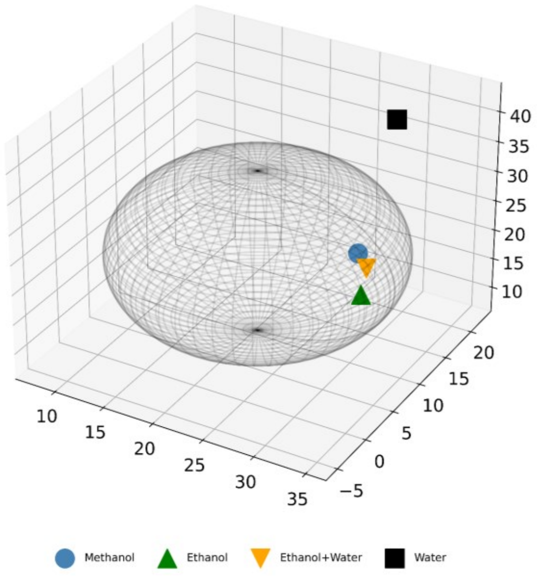

Figure S8. Hansen solubility parameters sphere for lignin using HSP values reported by Curvalo et al[10].

Table S3. HSP values for all solvents[7] and RED values calculated using Eqn S2 and S3.

Solvent system	δ_D	δ_P	δ_H	RED
Methanol	14.7	12.3	22.3	1.03
Ethanol	15.8	8.8	19.4	0.85
Ethanol+Water (85:15 v:v)	15.755	9.88	22.835	0.84
Water	15.5	16	42.3	1.83
Lignin[a]	21.42	8.57	21.80	

[a]Lignin HSP (($\delta_D, \delta_P, \delta_H$) is 21.42, 8.57, 21.80 and R_0 = 13.56) taken from Curvalo et al.[10]

Analysis of bulk solvation in ethanol+water mixtures

Since lignin has both polar and non-polar chain segments, to further explain the similar bulk solvation behavior for ethanol and ethanol+water observed in the MD simulations, we computed radial distribution functions (RDFs) between the methoxy, phenolic hydroxyl, aliphatic hydroxyl groups and aromatic ring of B1 lignin oligomer and ethanol and water molecules in ethanol+water, as well as in pure ethanol and pure water at 298 K (Figure S9). RDF analysis shows that in ethanol+water mixture, both ethanol and water molecules interact with the aliphatic hydroxyl groups and methoxy groups. Notably, water exhibits a stronger interaction with the aliphatic hydroxyl groups compared to ethanol, consistent with earlier reported preferential solvation trends observed in methanol+water mixture[58]. However, when comparing with pure solvents (Figure S9c, S9d), the interaction between the aliphatic hydroxyl group and water is weaker in pure water than in ethanol+water. This indicates the solvation environment in ethanol+water is most likely similar to pure ethanol, supporting more extended lignin conformation similar to those observed in pure ethanol (Figure 3 of the main text).

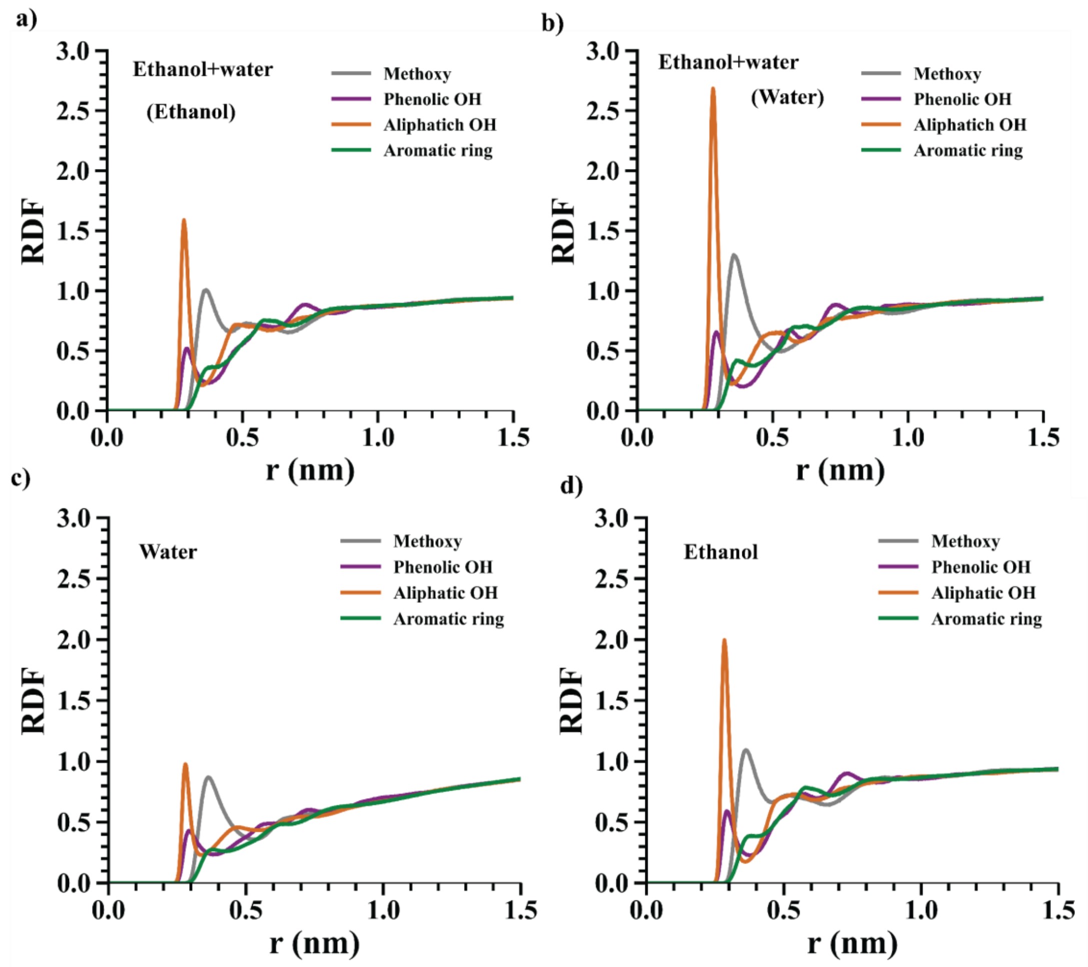

Figure S9. Radial distribution functions (RDFs) between the methoxy, phenolic OH , aliphatic OH groups and aromatic ring of B1 lignin oligomer with (a) ethanol ethanol+water and (b) water in ethanol+water mixture, (c) water and (d) ethanol at 298 K.

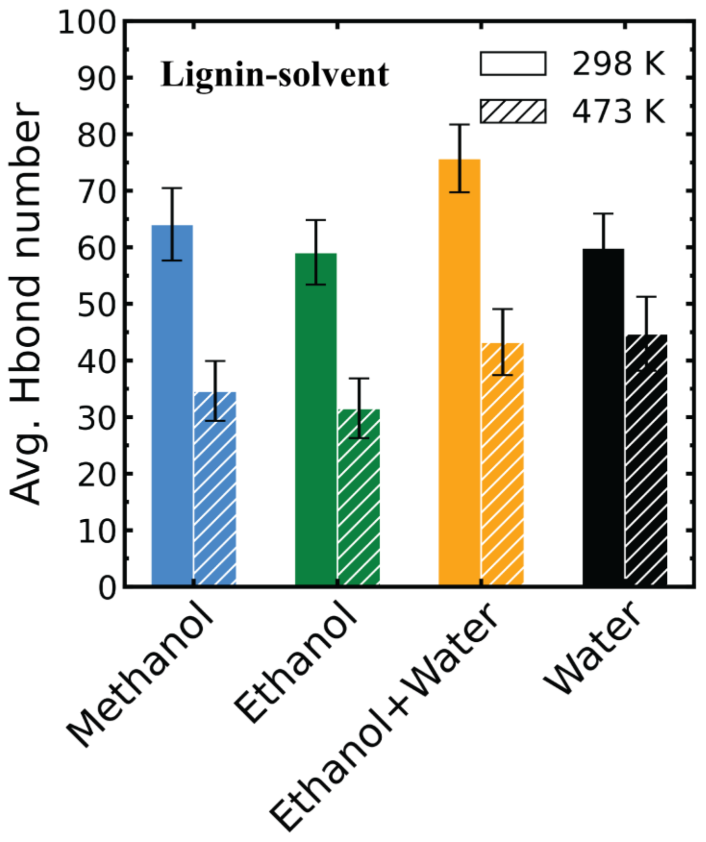

Figure S10. Time-averaged number of hydrogen bonds between the B1 lignin oligomer and different solvents at 298 K and 473 K.

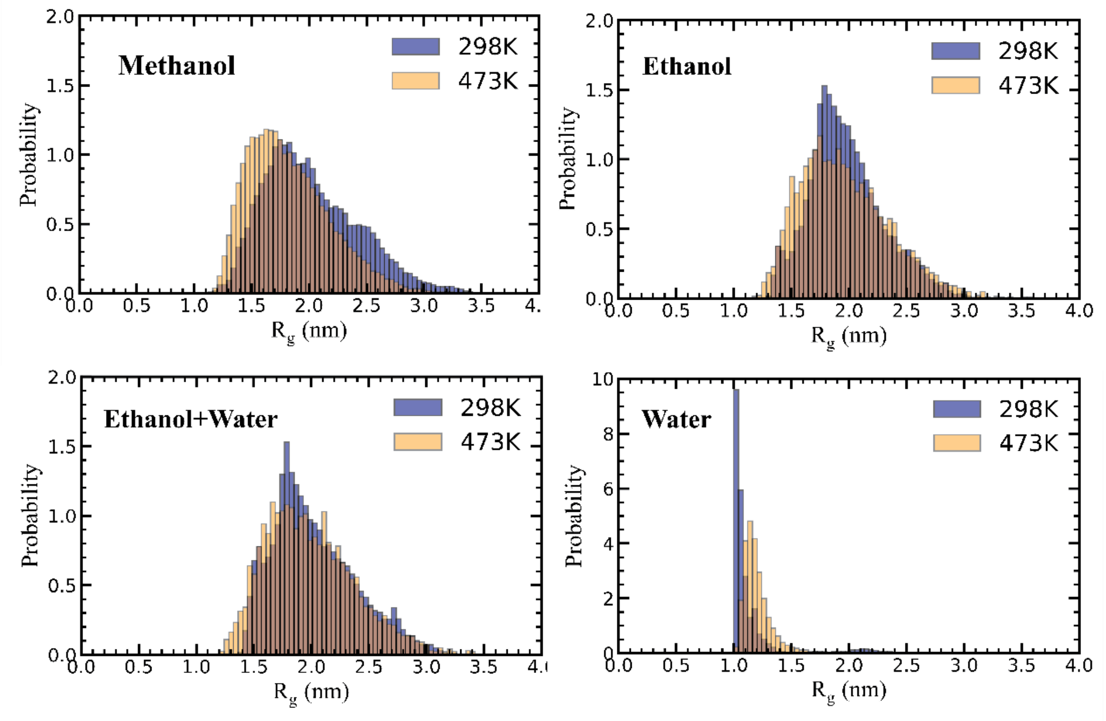

Figure S11. Histograms of R_g for the B1 oligomer in methanol, ethanol, ethanol+water and water at 298 K and 473 K.

Section 4.3 Simulation snapshots for lignin interactions with model surfaces

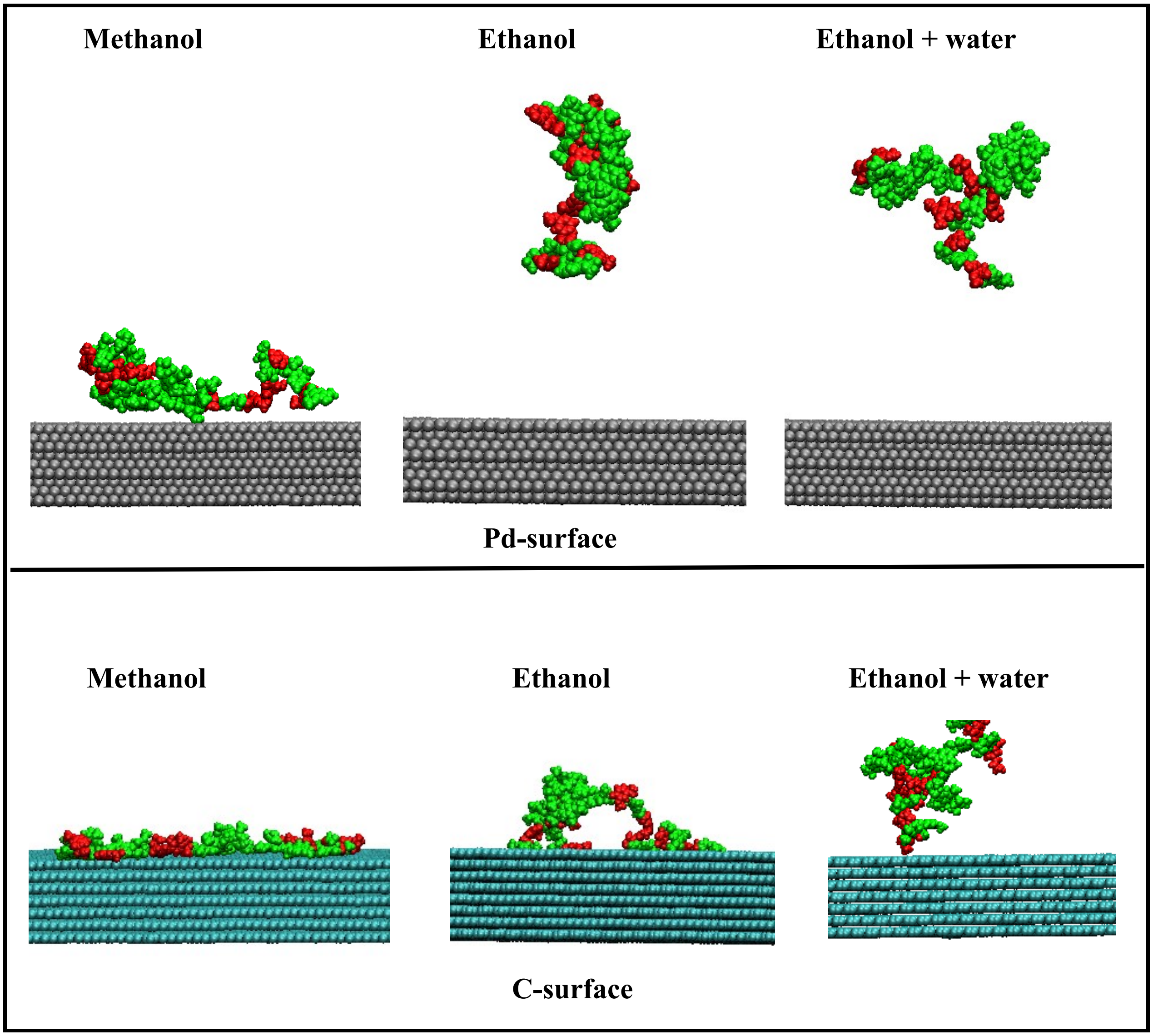

Figure S12 Simulation snapshots to illustrate interactions between lignin with Pd and C surfaces for (a) methanol, (b) ethanol and (c) ethanol+water at 298 K. Solvents are not shown for clarity.

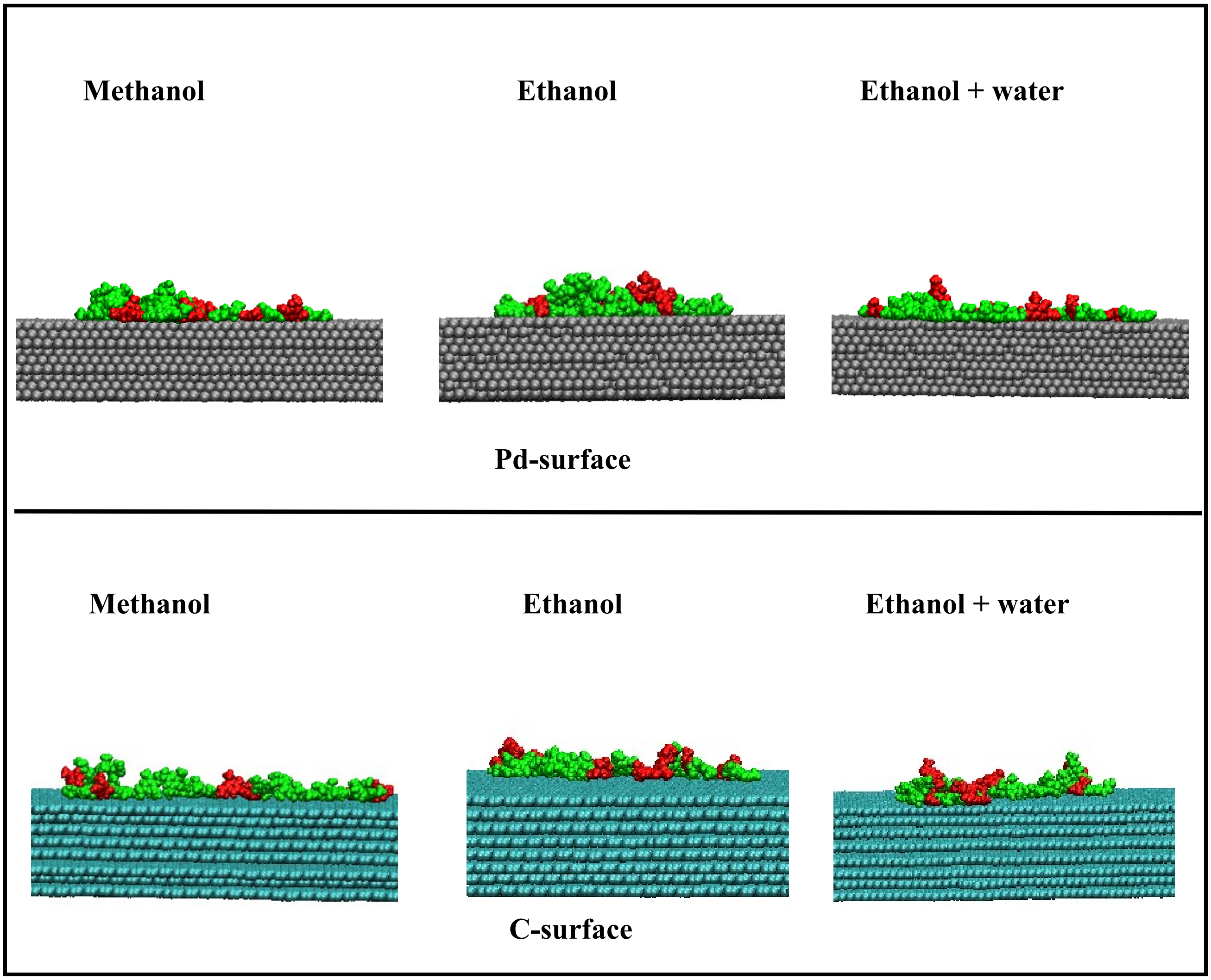

Figure S13. Simulation snapshots to illustrate interaction between lignin with Pd and C surfaces for (a) methanol, (b) ethanol and (c) ethanol+water at 473 K. Solvents are not shown for clarity.

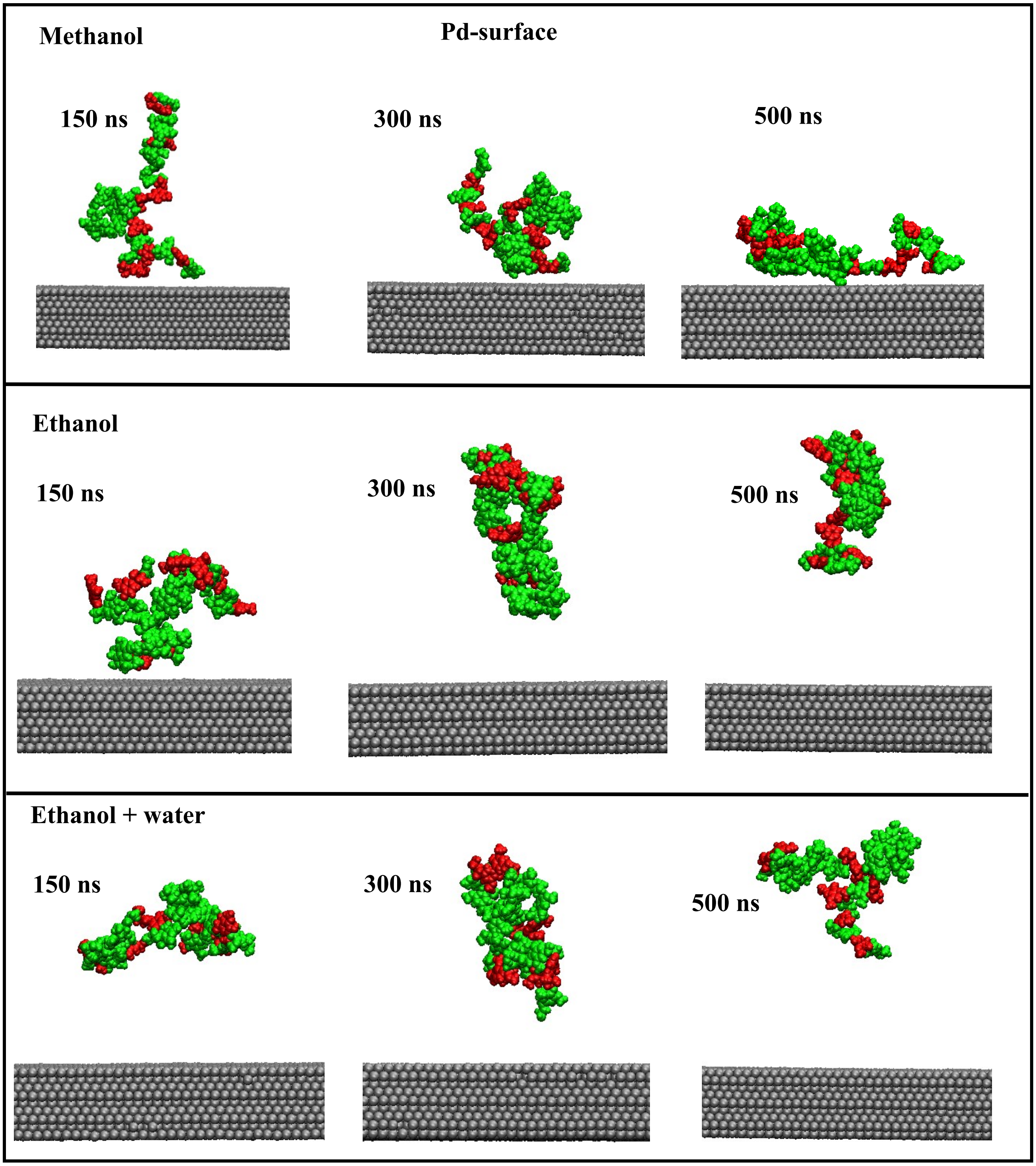

Figure S14. Simulation snapshots illustrating lignin-Pd surface interactions in methanol, ethanol and ethanol+water solvent at 298 K. Snapshots are extracted from various points of trajectories. Solvent molecules are not shown for clarity.

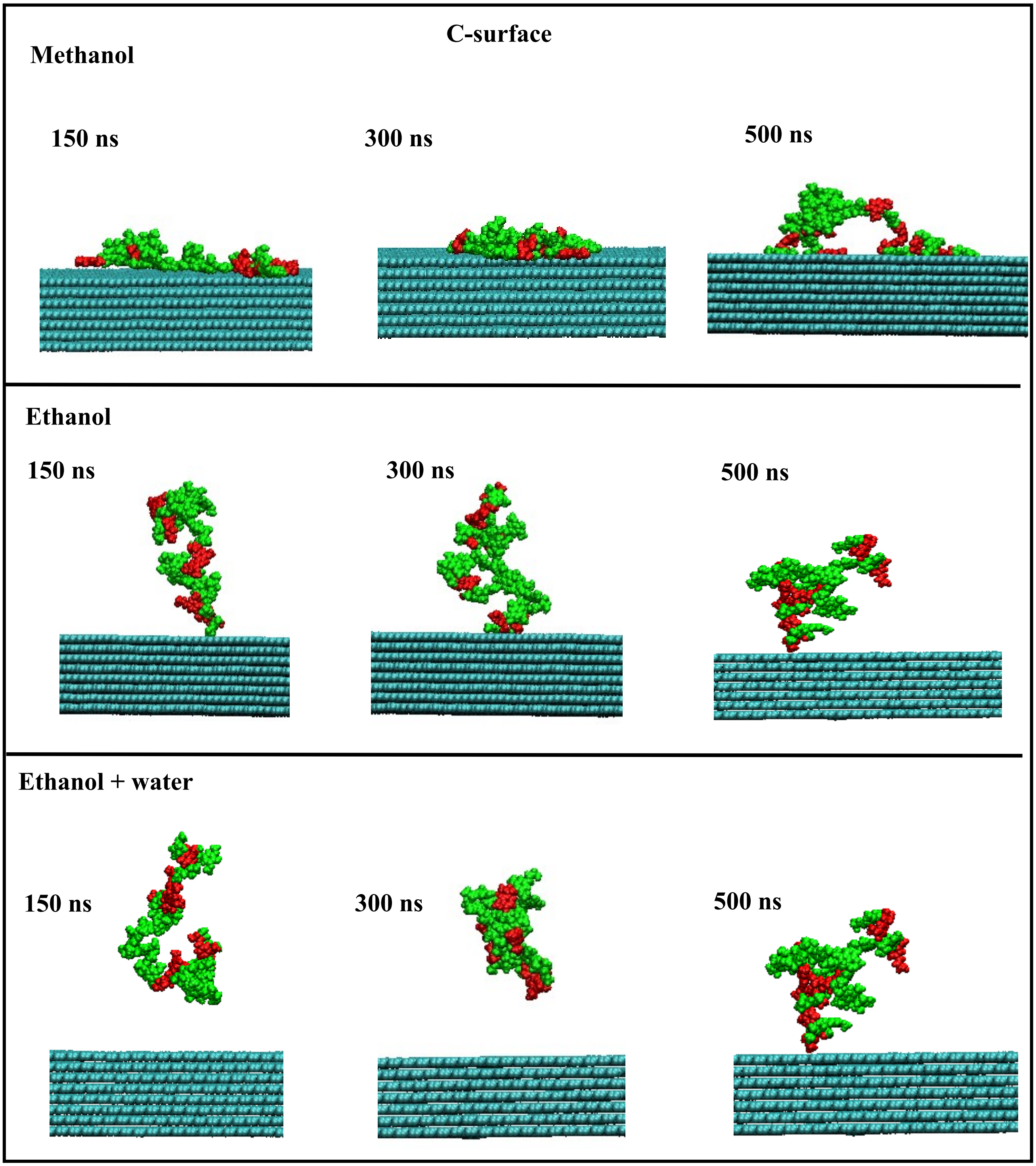

Figure S15. Simulation snapshots illustrating lignin-C surface interactions in methanol, ethanol and ethanol+water solvent at 298 K. Snapshots are extracted from various points of trajectories. Solvent molecules are not shown for clarity.

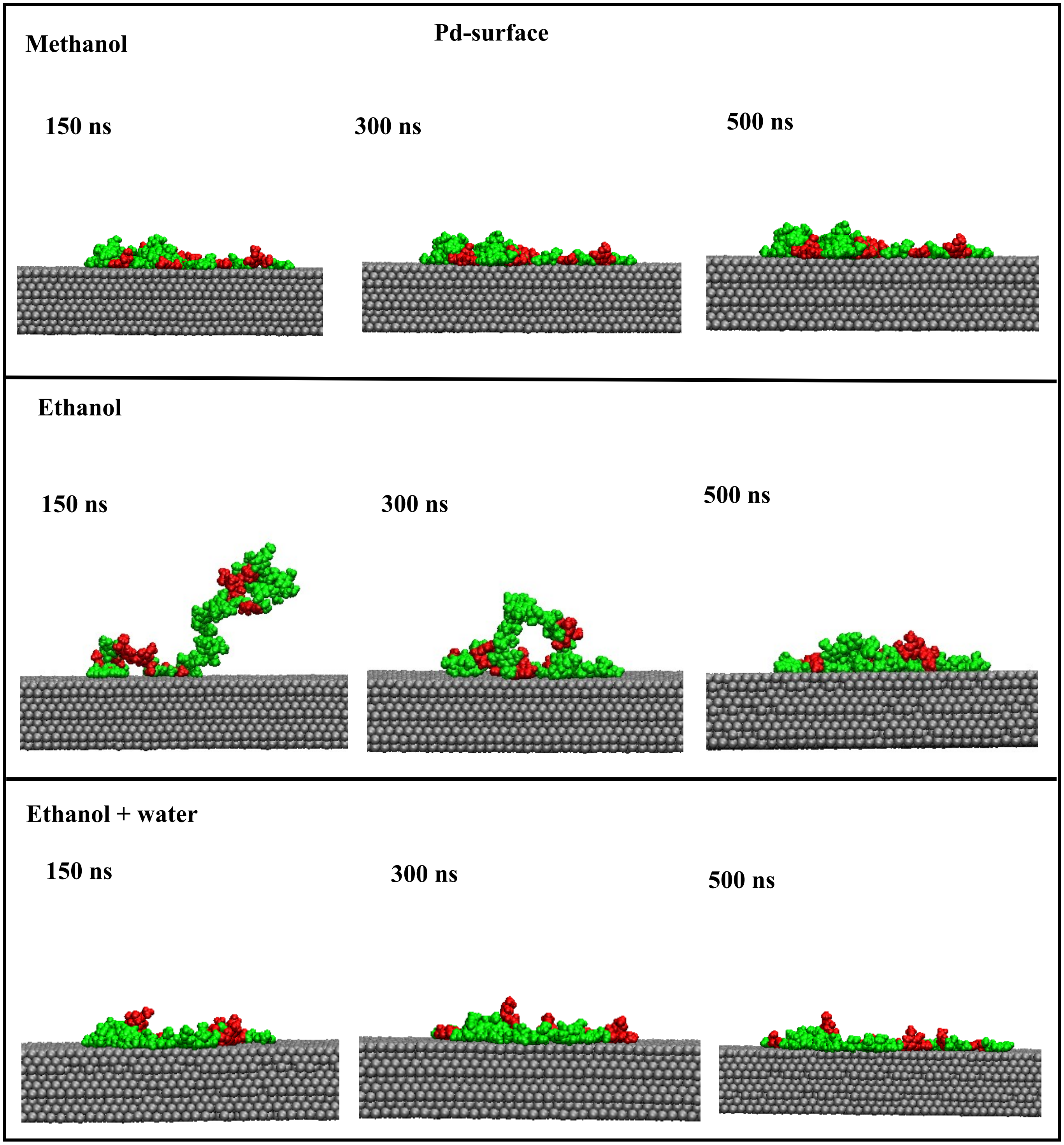

Figure S16. Simulation snapshots illustrating lignin-Pd surface interactions in methanol, ethanol and ethanol+water solvent at 473 K. Snapshots are extracted from various points of trajectories. Solvent molecules are not shown for clarity.

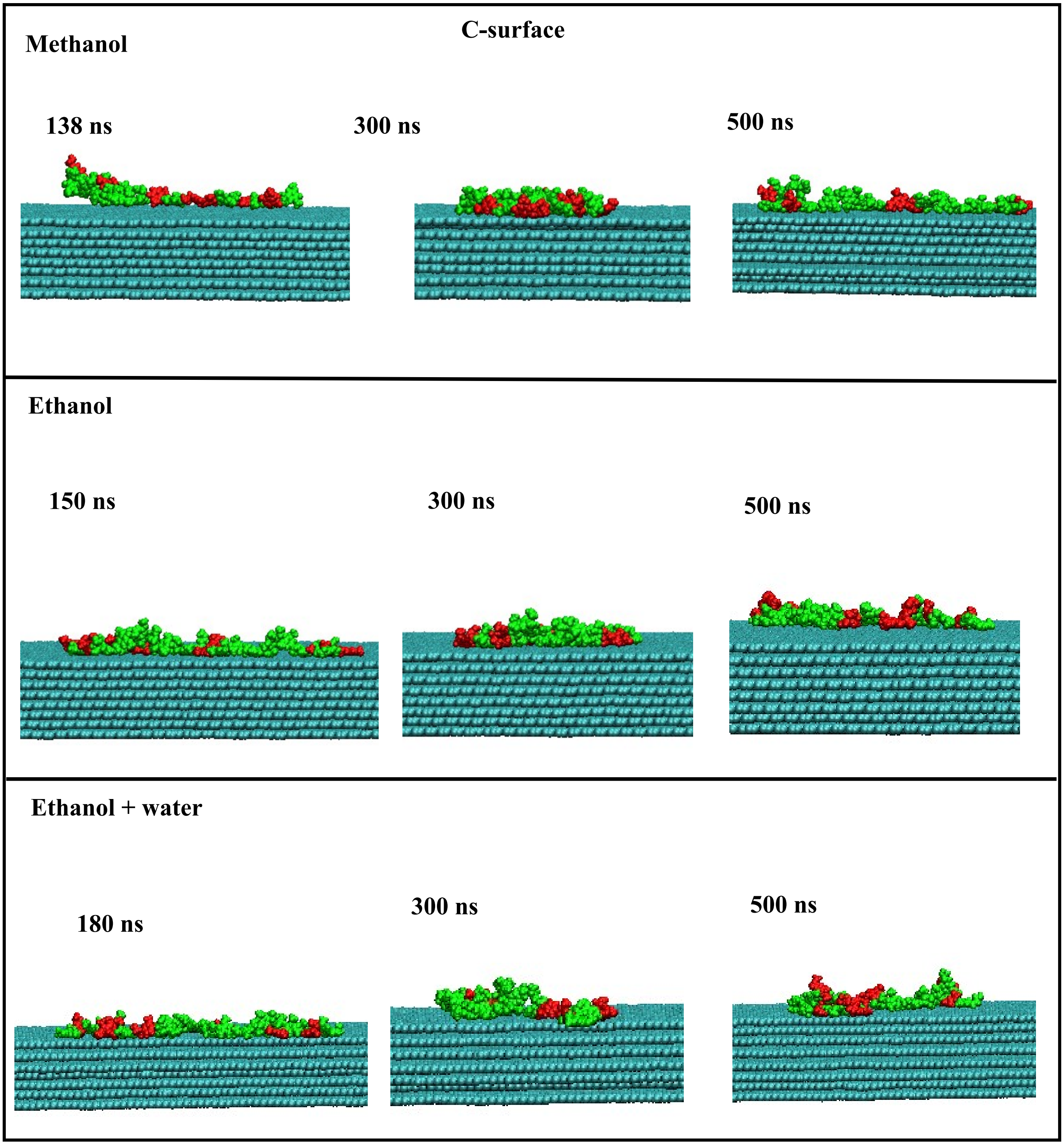

Figure S17. Simulation snapshots illustrating lignin-C surface interactions in methanol, ethanol and ethanol+water solvent at 473 K. Snapshots are extracted from various points of trajectories. Solvent molecules are not shown for clarity.

Table S4. Adsorption energies (E_{ads}) for lignin on Pd at 298 K and 473 K. The table includes the four energy components that contribute to E_{ads}. Errors are computed as described in Section S3. The table also includes the average adsorption energy ($E_{ads,avg}$) from two replica simulations.

Solvent	Energies	Replica 1	Replica 2	$E_{ads,avg}$
		T = 298 K		
Methanol	$\boldsymbol{E_{ads}}$ **(kJ/mol)**<br>E_i (kJ/mol)	28.0 ± 51.8<br>-1611500 ± 11.7 (E1)<br>456195 ± 18.2 (E2)<br>453013 ± 30.0 (E3)<br>-1614710 ± 36.3 (E4)	416.0 ± 40.3<br>-1611060 ± 16.5 (E1)<br>456134 ± 30.21 (E2)<br>453000 ± 13.16 (E3)<br>-1614610 ± 16.36 (E4)	222.0
Ethanol	$\boldsymbol{E_{ads}}$ **(kJ/mol)**<br>E_i (kJ/mol)	-2817.0 ± 307.9<br>-2386040 ± 80.5 (E1)<br>-323256 ± 27.6 (E2)<br>-338833 ± 19.18 (E3)<br>-2398800 ± 295.34 (E4)	-1094.0 ± 189.6<br>-2385140 ± 28.8 (E1)<br>-323924 ± 30.5 (E2)<br>-338838 ± 22.5 (E3)<br>-2398960 ± 183.54 (E4)	−1955.0
Ethanol+Water	$\boldsymbol{E_{ads}}$ **(kJ/mol)**<br>E_i (kJ/mol)	-2195.0 ± 389.2<br>-2560280 ± 189.7 (E1)<br>-492697 ± 38.43 (E2)<br>-502332 ± 33.74 (E3)<br>-2567720 ± 335.99 (E4)	-1169.0 ± 388.5<br>-2560140 ± 186.7 (E1)<br>-492693 ± 29.56 (E2)<br>-502272 ± 35.49 (E3)<br>-2568550 ± 337.57 (E4)	−1682.0
		T = 473 K		
Methanol	$\boldsymbol{E_{ads}}$ **(kJ/mol)**<br>E_i (kJ/mol)	1278.0 ± 87.11<br>-1379200 ± 23.5 (E1)<br>694577 ± 69.4 (E2)<br>694215 ± 23.4 (E3)<br>-1380840 ± 40.8 (E4)	477.0 ± 124.46<br>-1379640 ± 78.1 (E1)<br>695049 ± 45.6 (E2)<br>694326 ± 82.9 (E3)<br>-1380840 ± 20.6 (E4)	877.5
Ethanol	$\boldsymbol{E_{ads}}$ **(kJ/mol)**<br>E_i (kJ/mol)	-209.0 ± 102.9<br>-2148600 ± 22.3 (E1)<br>-96589 ± 81.7 (E2)<br>-109628 ± 46.5 (E3)<br>-2161430 ± 35.6 (E4)	678.0 ± 199.7<br>-2148170 ± 39.5 (E1)<br>-97029 ± 192.6 (E2)<br>-109591 ± 7.5 (E3)<br>-2161410 ± 34.4 (E4)	234.5
Ethanol+Water	$\boldsymbol{E_{ads}}$ **(kJ/mol)**<br>E_i (kJ/mol)	-162.0 ± 101.03<br>-2281430 ± 39.5 (E1)<br>-231305 ± 71.2 (E2)<br>-242700 ± 49.7 (E3)<br>-2292710 ± 33.3 (E4)	-69.0 ± 72.64<br>-2281430 ± 39.9 (E1)<br>-231329 ± 27.2 (E2)<br>-242680 ± 15.1 (E3)<br>-2292720 ± 52.1 (E4)	−115.5

Table S5. Adsorption energies (E_{ads}) for lignin on C at 298 K and 473 K. The table includes the four energy components that contribute to E_{ads}. Errors are computed as described in Section S3. The table also includes the average adsorption energy ($E_{ads,avg}$) from two replica simulations.

Solvents	Energies	Replica 1	Replica 2	$E_{ads,avg}$
T = 298 K				
Methanol	$\boldsymbol{E_{ads}}$ **(kJ/mol)**<br>E_i (kJ/mol)	16749.0 ± 45.1<br>906442 ± 12.8 (E1)<br>456266 ± 37.5 (E2)<br>470834 ± 28.8 (E3)<br>904261 ± 26.3 (E4)	16695.0 ± 73.0<br>906528 ± 17.9 (E1)<br>456364 ± 20.5 (E2)<br>470786 ± 47.1 (E3)<br>904255 ± 48.7 (E4)	16722.0
Ethanol	$\boldsymbol{E_{ads}}$ **(kJ/mol)**<br>E_i (kJ/mol)	-670.0 ± 30.5<br>102267 ± 14.8 (E1)<br>-340011 ± 12.7 (E2)<br>-355187 ± 15.3 (E3)<br>87761 ± 17.8 (E4)	-640.1 ± 59.1<br>102275 ± 18.1 (E1)<br>-339978 ± 30.1 (E2)<br>-355187 ± 18.9 (E3)<br>87761 ± 43.6 (E4)	−655.0
Ethanol+Water	$\boldsymbol{E_{ads}}$ **(kJ/mol)**<br>E_i (kJ/mol)	-21891.0 ± 118.2<br>-92532 ± 12.3 (E1)<br>-510379 ± 25.6 (E2)<br>-519886 ± 76.6 (E3)<br>-80148 ± 85.3 (E4)	-21103.0 ± 95.6<br>-92537 ± 52.9 (E1)<br>-510408 ± 30.9 (E2)<br>-519905 ± 50.7 (E3)<br>-80931 ± 52.9 (E4)	−21497.0
T = 473 K				
Methanol	$\boldsymbol{E_{ads}}$ **(kJ/mol)**<br>E_i (kJ/mol)	351.0 ± 265.1<br>1180470 ± 28.4 (E1)<br>722264 ± 132.5 (E2)<br>723809 ± 218.6 (E3)<br>1181280 ± 64.1 (E4)	825.0 ± 150.1<br>1180870 ± 16.2 (E1)<br>722741 ± 65.2 (E2)<br>724006 ± 92.6 (E3)<br>1181310 ± 97.1 (E4)	588.0
Ethanol	$\boldsymbol{E_{ads}}$ **(kJ/mol)**<br>E_i (kJ/mol)	-1863.0 ± 72.6<br>384803 ± 34.2 (E1)<br>-101948 ± 39.2 (E2)<br>-114869 ± 27.4 (E3)<br>371745 ± 42.6 (E4)	-1574.0 ± 123.4<br>384746 ± 45.7 (E1)<br>-101966 ± 91.1 (E2)<br>-114863 ± 44.6 (E3)<br>373423 ± 53.6 (E4)	−1718.5
Ethanol+Water	$\boldsymbol{E_{ads}}$ **(kJ/mol)**<br>E_i (kJ/mol)	-12876.0 ± 106.2<br>238726 ± 12.5 (E1)<br>-239874 ± 41.0 (E2)<br>-251296 ± 24.4 (E3)<br>240180 ± 94.0 (E4)	-11594.0 ± 163.0<br>239793 ± 36.4 (E1)<br>-239874 ± 54.7 (E2)<br>-251130 ± 48.6 (E3)<br>239958 ± 141.1 (E4)	−12235.0

Table S6. Difference in adsorption energy components, $E_4 - E_3$ (where E_4 is the average energy of the solvent-surface system and E_3 is the average energy of the pure solvent system) for Pd at 298 K and 473 K. Errors are computed as described in Section S3. Numerical values for E_4 and E_3 are provided in Table S3. We subtracted the Lennard-Jones interaction energy for Pd-Pd from E_4, ensuring that it includes only solvent-solvent and solvent-surface interactions.

Solvents	Replica 1* $E_4 - E_3$ (kJ/mol)	Replica 2* $E_4 - E_3$ (kJ/mol)
T = 298 K		
Methanol	-113133 ± 47.1	-113020 ± 21.0
Ethanol	-105377 ± 295.9	-105532 ± 184.9
Ethanol+Water	-110798 ± 333.7	-111688 ± 339.4
T = 473 K		
Methanol	-165035 ± 47.0	-165146 ± 85.4
Ethanol	-141782 ± 58.6	-141799 ± 35.2
Ethanol+Water	-139990 ± 59.8	-140020 ± 54.2

*To obtain Lennard-Jones interaction energy for Pd-Pd, we performed *NVT* simulations of the Pd surface in vacuum at both temperatures.

Table S7. Difference in adsorption energy components, $E_4 - E_3$ (where E_4 is the average energy of the solvent-surface system and E_3 is the average energy of the pure solvent system) for C at 298 K and 473 K. Errors are computed as described in Section S3. Numerical values for E_4 and E_3 are provided in Table S4. We subtracted the Lennard-Jones interaction energy for C-C from E_4, ensuring that it includes only solvent-solvent and solvent-surface interactions.

Solvents	Replica 1* $E_4 - E_3$ (kJ/mol)	Replica 2* $E_4 - E_3$ (kJ/mol)
T = 298 K		
Methanol	30288 ± 39.0	30330 ± 67.7
Ethanol	39809 ± 23.5	39809 ± 47.5
Ethanol+Water	36599 ± 114.6	35835 ± 73.3
T = 473 K		
Methanol	52757 ± 227.8	52590 ± 134.2
Ethanol	81900 ± 50.6	83572 ± 69.7
Ethanol+Water	86762 ± 97.1	86374 ± 149.2

*To obtain Lennard-Jones interaction energy for C-C, we performed *NVT* simulations of the C surface in vacuum at both temperatures.

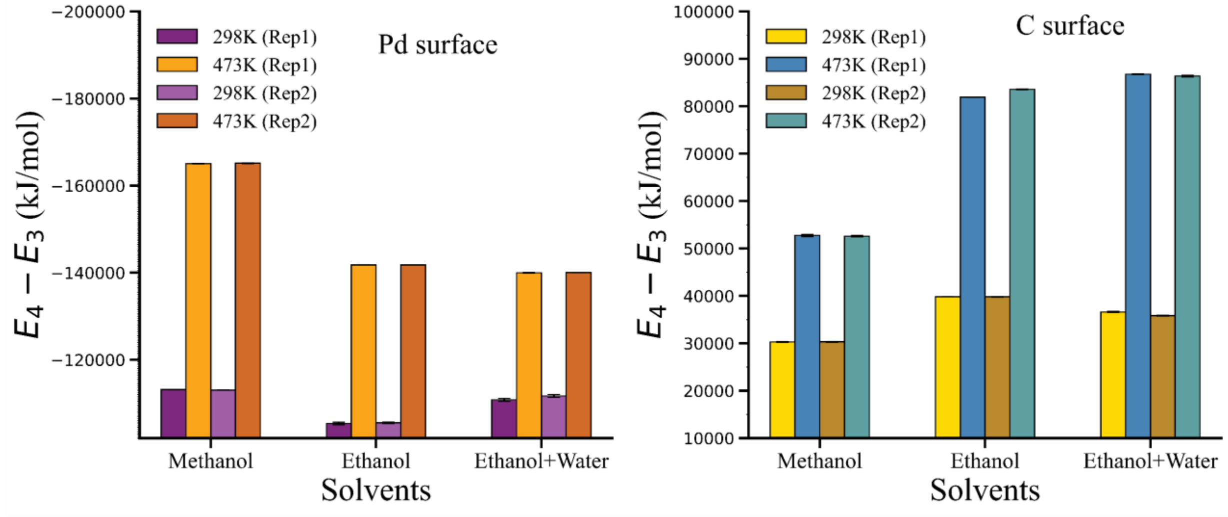

Figure S18. Difference in adsorption energy components, $E_4 - E_3$ (where E_4 is the average energy of the solvent-surface system and E_3 is the average energy of the pure solvent system) for Pd and C surfaces at 298 K and 473 K. Errors are computed as described in Section S3. Numerical values for $E_4 - E_3$ for Pd and C surfaces are provided in Table S5 and S6 respectively.

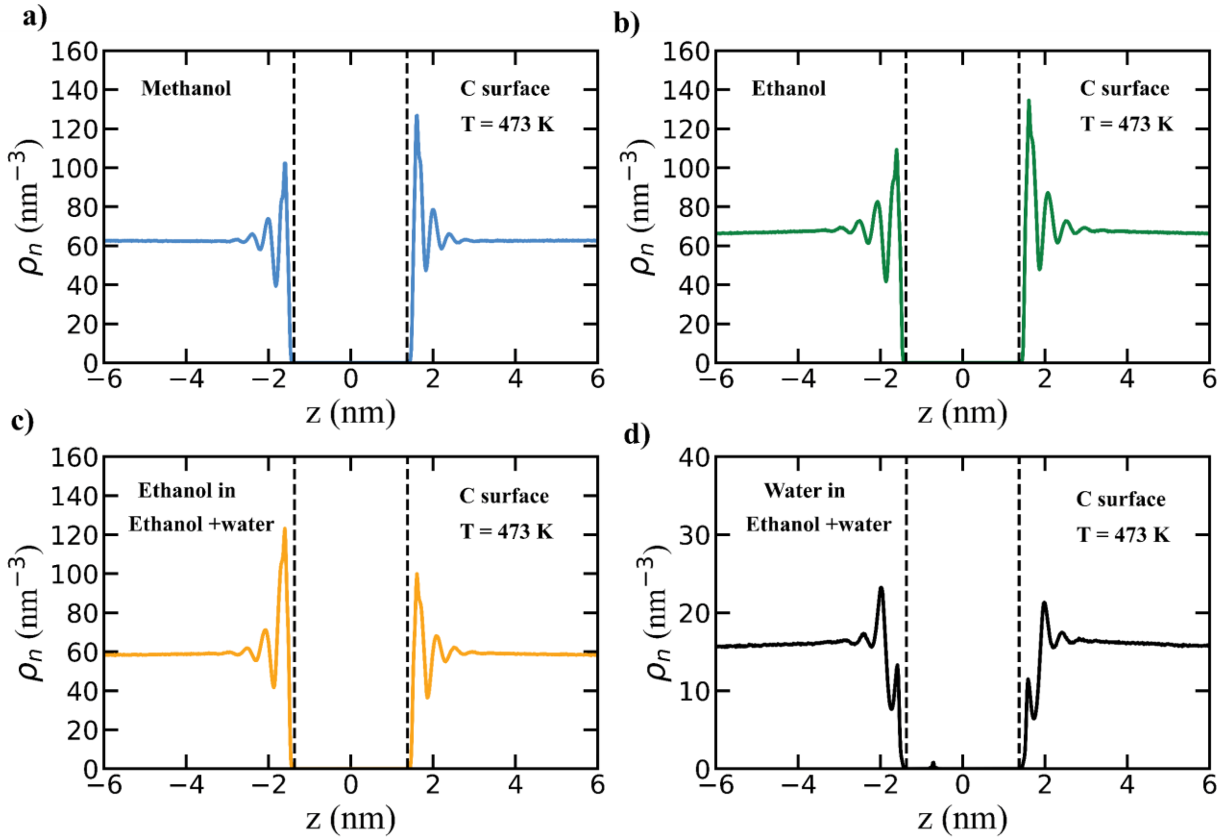

Figure S19. Number density (ρ_n) profiles for solvents along z-axis for pure methanol (a), pure ethanol (b), and ethanol (c) and water (d) in the ethanol+water mixture in the presence of the C surface at 473 K. Black dashed lines indicate the C surface, which presents two surfaces that interact with the solvent due to the periodic boundary conditions. The lower solvent density on one of the two surfaces is attributed to the displacement of solvent molecules by adsorbed lignin.

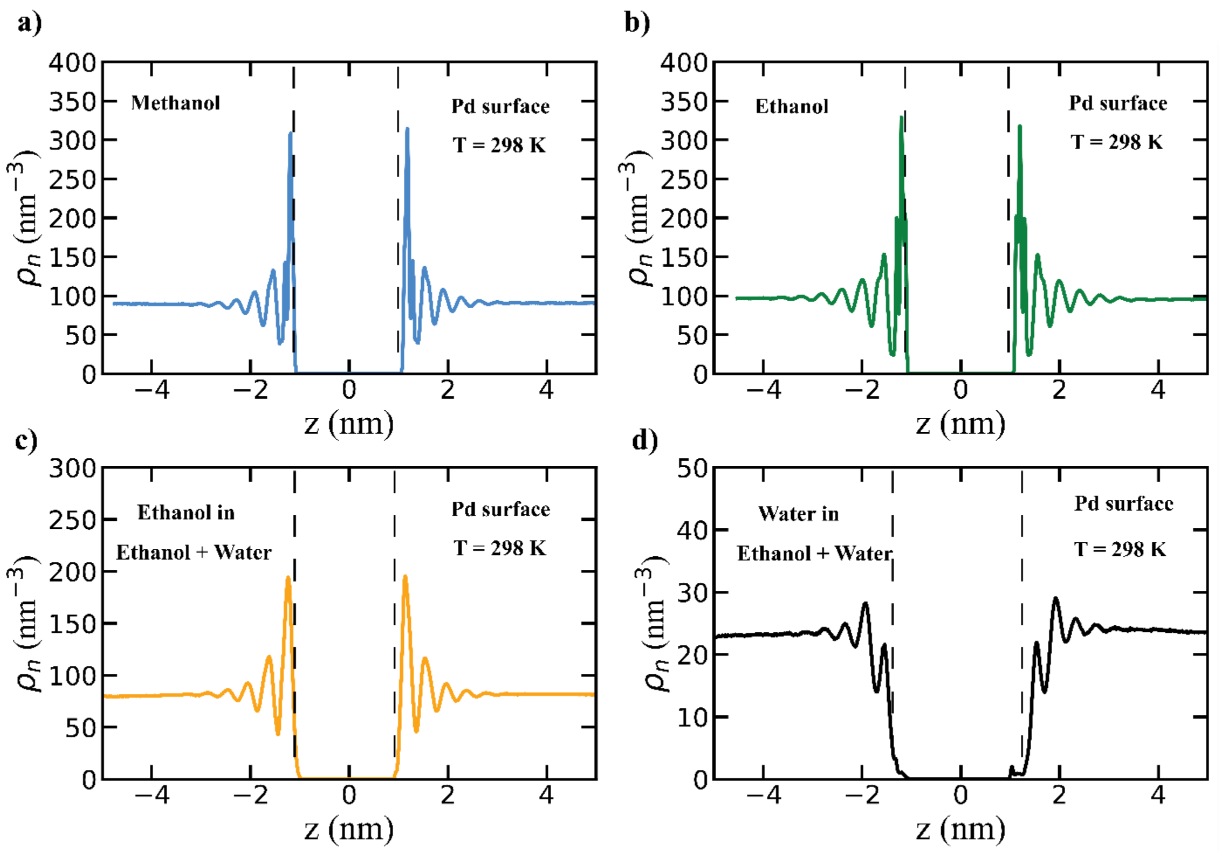

Figure S20. Number density (ρ_N) profiles for solvents along z-axis for pure methanol (a), pure ethanol (b), and ethanol (c) and water (d) for the ethanol+water solvent mixture in the presence of the Pd surface at 298 K. Black dashed lines indicate the Pd surface, which presents two surfaces that interact with the solvent due to the periodic boundary conditions

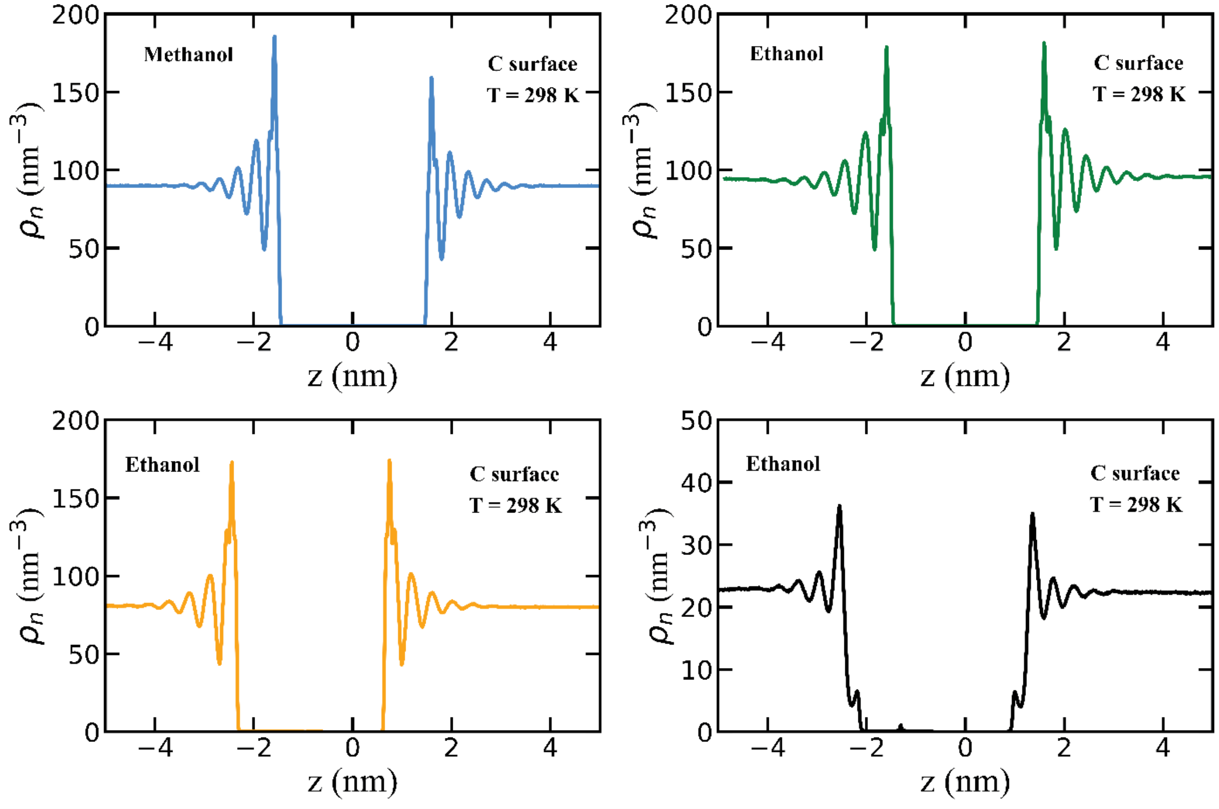

Figure S21. Number density (ρ_N) profiles for solvents along z-axis for pure methanol (a), pure ethanol (b), and ethanol (c) and water (d) for the ethanol+water solvent mixture in the presence of the C surface at 298 K. Black dashed lines indicate the C surface, which presents two surfaces that interact with the solvent due to the periodic boundary conditions

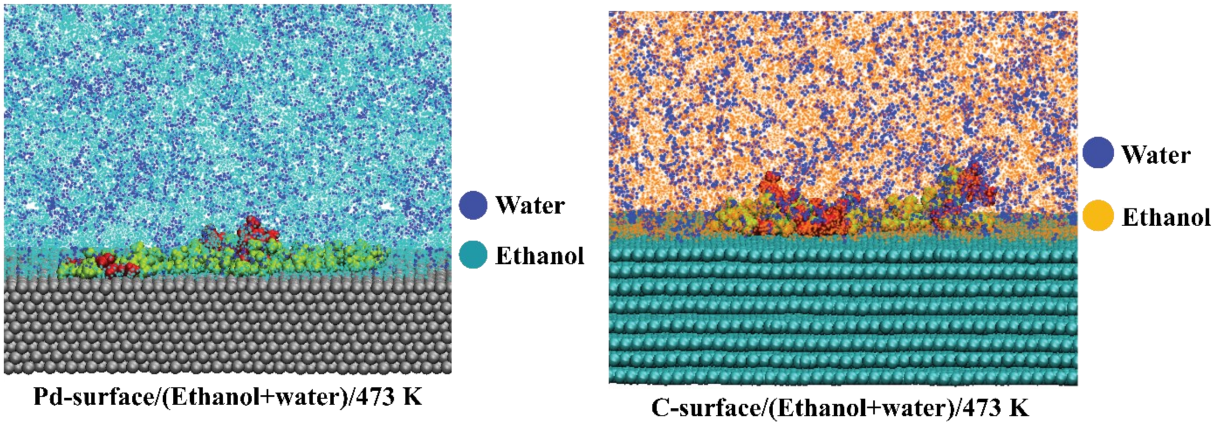

Figure S22. Representative simulation snapshots to illustrate solvent density in the interfacial region. Snapshots are shown for the lignin oligomer, Pd/C surface and ethanol+water at 473 K.

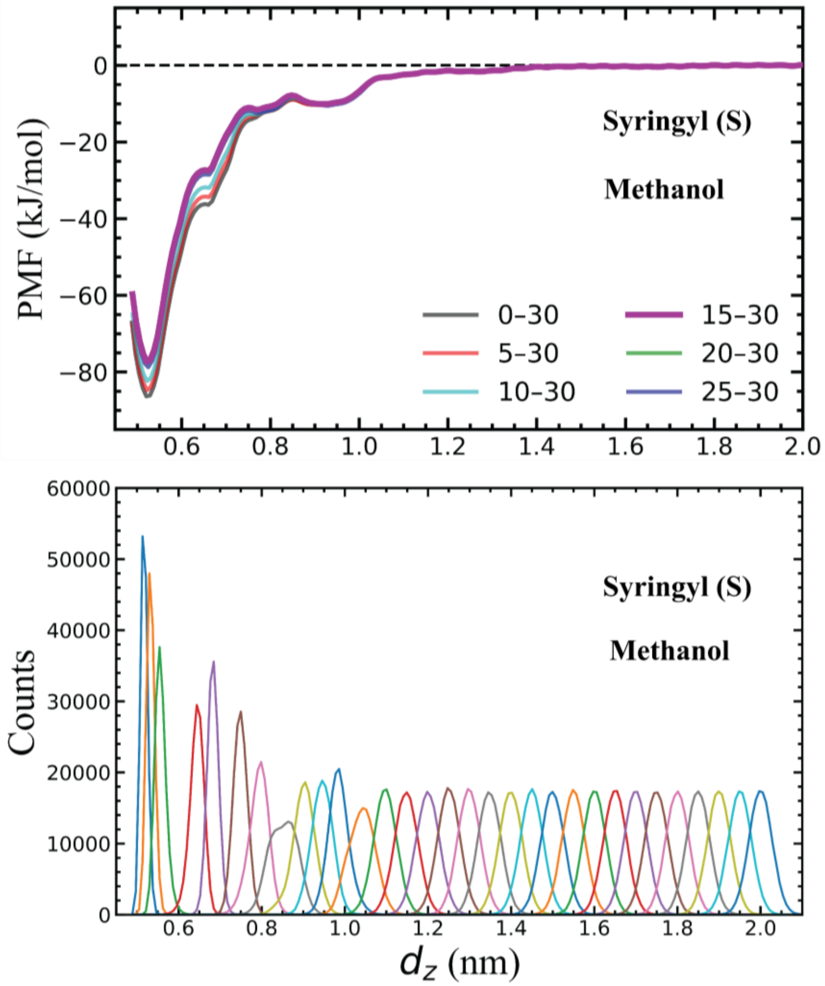

Figure S23. Convergence of potential of mean force (PMF) profiles for the syringyl (S) monomer in methanol. The top panel shows PMF profiles computed for different time intervals (in ns) out of the complete 30 ns trajectory. The final PMF used in the analysis was obtained by using the 15-30 ns interval. The bottom panel displays the corresponding histograms as a function of the reaction coordinate, confirming sufficient sampling across umbrella windows.

Table S8. Number of solvent molecules near the Pd surface (within 0.6 nm) at 473 K, in the presence and absence of adsorbed syringyl (S) and guaiacyl (G) monomers. The number of solvent molecules displaced due to monomer adsorption is also reported. Both values were obtained following the approach used for quantifying solvent displacement by lignin oligomers in the main text. For ethanol+water, the number of water molecules is shown in parentheses, while the number outside parentheses corresponds to ethanol. All values were computed from the umbrella sampling windows with 0.5 nm $\leq d_z \leq$ 0.6 nm corresponding to the adsorbed state of the monomer.

Syringyl (S) monomer			
Solvents	In the presence of the monomer	In the absence of the monomer	Number of solvent molecules displaced
Methanol	427	403	24
Ethanol	424	412	12
Ethanol+water	367 (47)	347 (27)	20 (20)
Guaiacyl (G) monomer			
Solvents	In the presence of the monomer	In the absence of the monomer	Number of solvent molecules displaced
Methanol	431	410	21
Ethanol	490	436	60
Ethanol+water	367 (101)	349 (97)	17 (4)

References

(1) Choi, Y. K.; Kern, N. R.; Kim, S.; Kanhaiya, K.; Afshar, Y.; Jeon, S. H.; Jo, S.; Brooks, B. R.; Lee, J.; Tadmor, E. B.; Heinz, H.; Im, W. CHARMM-GUI Nanomaterial Modeler for Modeling and Simulation of Nanomaterial Systems. *J. Chem. Theory Comput.* **2022**, *18* (1), 479–493. https://doi.org/10.1021/acs.jctc.1c00996.

(2) Jo, S.; Kim, T.; Iyer, V. G.; Im, W. CHARMM-GUI: A Web-Based Graphical User Interface for CHARMM. *Journal of Computational Chemistry* **2008**, *29* (11), 1859–1865. https://doi.org/10.1002/jcc.20945.

(3) Heinz, H.; Lin, T.-J.; Kishore Mishra, R.; Emami, F. S. Thermodynamically Consistent Force Fields for the Assembly of Inorganic, Organic, and Biological Nanostructures: The INTERFACE Force Field. *Langmuir* **2013**, *29* (6), 1754–1765. https://doi.org/10.1021/la3038846.

(4) Heinz, H. Computational Screening of Biomolecular Adsorption and Self-Assembly on Nanoscale Surfaces. *Journal of Computational Chemistry* **2010**, *31* (7), 1564–1568. https://doi.org/10.1002/jcc.21421.

(5) Heinz, H.; Farmer, B. L.; Pandey, R. B.; Slocik, J. M.; Patnaik, S. S.; Pachter, R.; Naik, R. R. Nature of Molecular Interactions of Peptides with Gold, Palladium, and Pd−Au Bimetal Surfaces in Aqueous Solution. *J. Am. Chem. Soc.* **2009**, *131* (28), 9704–9714. https://doi.org/10.1021/ja900531f.

(6) Flyvbjerg, H.; Petersen, H. G. Error Estimates on Averages of Correlated Data. *The Journal of Chemical Physics* **1989**, *91* (1), 461–466. https://doi.org/10.1063/1.457480.

(7) Hansen, C. M. *Hansen Solubility Parameters: A User's Handbook, Second Edition*, 2nd ed.; CRC Press: Boca Raton, 2007. https://doi.org/10.1201/9781420006834.

(8) Zhang, Q.; Tan, X.; Wang, W.; Yu, Q.; Wang, Q.; Miao, C.; Guo, Y.; Zhuang, X.; Yuan, Z. Screening Solvents Based on Hansen Solubility Parameter Theory To Depolymerize Lignocellulosic Biomass Efficiently under Low Temperature. *ACS Sustainable Chem. Eng.* **2019**, *7* (9), 8678–8686. https://doi.org/10.1021/acssuschemeng.9b00494.

(9) Passoni, V.; Scarica, C.; Levi, M.; Turri, S.; Griffini, G. Fractionation of Industrial Softwood Kraft Lignin: Solvent Selection as a Tool for Tailored Material Properties. *ACS Sustainable Chem. Eng.* **2016**, *4* (4), 2232–2242. https://doi.org/10.1021/acssuschemeng.5b01722.

(10) Novo, L. P.; Curvelo, A. A. S. Hansen Solubility Parameters: A Tool for Solvent Selection for Organosolv Delignification. *Ind. Eng. Chem. Res.* **2019**, *58* (31), 14520–14527. https://doi.org/10.1021/acs.iecr.9b00875.
